# Nudel and FAK as Antagonizing Strength Modulators of Nascent Adhesions through Paxillin

**DOI:** 10.1371/journal.pbio.1000116

**Published:** 2009-05-26

**Authors:** Yongli Shan, Lihou Yu, Yan Li, Youdong Pan, Qiangge Zhang, Fubin Wang, Jianfeng Chen, Xueliang Zhu

**Affiliations:** Laboratory of Molecular Cell Biology, Institute of Biochemistry and Cell Biology, Shanghai Institutes for Biological Sciences, Chinese Academy of Sciences, Shanghai, China; National Institute of Dental and Craniofacial Research, United States of America

## Abstract

Adhesion and detachment are coordinated critical steps during cell migration. Conceptually, efficient migration requires both effective stabilization of membrane protrusions at the leading edge via nascent adhesions and their successful persistence during retraction of the trailing side via disruption of focal adhesions. As nascent adhesions are much smaller in size than focal adhesions, they are expected to exhibit a stronger adhesivity in order to achieve the coordination between cell front and back. Here, we show that Nudel knockdown by interference RNA (RNAi) resulted in cell edge shrinkage due to poor adhesions of membrane protrusions. Nudel bound to paxillin, a scaffold protein of focal contacts, and colocalized with it in areas of active membrane protrusions, presumably at nascent adhesions. The Nudel-paxillin interaction was disrupted by focal adhesion kinase (FAK) in a paxillin-binding–dependent manner. Forced localization of Nudel in all focal contacts by fusing it to paxillin markedly strengthened their adhesivity, whereas overexpression of structurally activated FAK or any paxillin-binding FAK mutant lacking the N-terminal autoinhibitory domain caused cell edge shrinkage. These results suggest a novel mechanism for selective reinforcement of nascent adhesions via interplays of Nudel and FAK with paxillin to facilitate cell migration.

## Abstract 2

Competition for binding to the cellular protein paxillin by the proteins Nudel and focal adhesion kinase is important for the proper regulation of cell adhesion and migration.

## Author Summary

Cell migration is an essential process in both single-cell and multicellular organisms. In higher animals, cell migration is important for many biological processes, including embryonic development, the immune response, and wound healing. Cancer cell invasion into healthy tissues occurs as a result of inappropriate cell migration. As can be easily visualized when cultured in the lab, mammalian cells attach to surfaces through focal adhesions, cellular structures characterized by complexes of the transmembrane protein integrin and intracellular proteins including paxillin and focal adhesion kinase (FAK). In order for cells to move, they must coordinate two processes: extension of the front edge of the cell and retraction of the back edge. To accomplish this, a cell first protrudes membranous structures from the front edge and then establishes adhesion structures known as nascent adhesions to hold the extensions in place. At the same time, the focal adhesions that hold a cell in place must be disrupted in order for the back edge of the cell to retract. Here, we show that a protein called Nudel is enriched at the front edge of moving cells, where it interacts with paxillin but is not detected in focal adhesions. We further show that the focal adhesion protein FAK is able to abolish the Nudel-paxillin interaction, leading to repression of the formation of nascent adhesions and to the loss of cell extensions. We therefore propose a model in which modulation of paxillin interactions in nascent adhesions and in focal adhesions is critical for coordinated cell movement: the Nudel-paxillin interaction enhances the strength of nascent adhesions to promote the attachment of membrane protrusions at the front edge of the cell, whereas FAK prevents the Nudel-paxillin interaction in focal adhesions in order to facilitate retraction of the back edge of the cell.

## Introduction

In order to achieve efficient migration, cell adhesion and detachment must be properly coordinated. Cells attach to the substratum via punctate focal contacts (FCs). FCs contain integrin family members of transmembrane receptors and a variety of intracellular “adhesion” proteins and function to connect the extracellular matrix (ECM) to the actin cytoskeleton [1],[2]. During cell migration, membrane protrusions at the leading edge are triggered after activation of the Rho-family small GTPases Cdc42 and Rac1 [3]. Activated integrin dimers situated at the tip of protrusions then search for and bind to their ligands in the ECM to form nascent adhesions [4]. Nascent adhesions can mature into focal complexes (FXs), which are located mainly at the base of lamellipodium [5],[6]. FXs associate with branched F-actin and are thought to facilitate the propulsive effect of the lamellipodium. Some FXs then further evolve into the largest form of FC, namely focal adhesion (FA). FAs associate with the termini of F-actin bundles, or stress fibers, which provide cells with contractile forces [1],[6]–[8].

It is known that only moderate concentrations of the ECM are associated with maximal cell motility [9],[10]. Conceptually, fast migration would require efficient adhesion of leading-edge protrusions and rapid retraction of the trailing side [10],[11]. These two requirements could be satisfied if nascent adhesion sites exhibit stronger ECM-binding strengths than adhesion sites in FXs as well as FAs. Stronger adhesions at nascent sites would both promote the stabilization of membrane protrusions and facilitate persistency of the leading edge during cell retraction to allow efficient forward movement. In fact, tension on each contact site within FAs, which are caps of stress fibers [1],[7], is relatively constant in a cell [12]. Therefore, smaller FAs can only transmit weaker traction forces [12]. In contrast, compared to FAs, nascent adhesions, despite their submicroscopic sizes, have been shown to transmit stronger traction forces [13]. This would physically require a stronger integrin-ECM interaction at nascent adhesion sites than at adhesion sites of FXs and FAs. Whether mechanical strengths of different adhesion sites are indeed modulated and the underlying molecular mechanism(s), however, remain unclear.

FCs are dynamic structures. They are assembled through a hierarchical process. Paxillin and talin are believed to bind directly to integrin at adhesion sites [2]. Other proteins such as vinculin and focal adhesion kinase (FAK) are then recruited to form dot-like FXs, whereas FA formation is accompanied by the appearance of zyxin [6],[14]. FAK can be activated by multiple signaling pathways and is crucial for FC dynamics and membrane protrusion [2]. Its FA-targeting (FAT) domain, located at the C-terminus, interacts with talin and the LD domains of paxillin [2],[15]. In addition to assembly, FCs are subjected to dynamic disassembly as well [7]. Both nascent adhesion sites and FXs can be rapidly disassembled if they failed to evolve [6],[14]. FAs are relatively long-lived. Their disassembly often occurs at the trailing side of migrating cells. Moreover, FA formation can be promoted by internal and external tensions [12],[16]–[18]. Tensions on stress fibers can also lead to a net disassembly of distal adhesion sites and assembly of proximal sites, resulting in centripetal movement of FAs [19].

Mammalian Nudel (also named Ndel1) and Lis1 are essential for cell viability [20],[21] and for functions of the microtubule (MT)-based, minus end–directed motor cytoplasmic dynein in diverse processes including mitosis, neuronal migration, and intracellular transport [20],[22]–[27]. In addition, Nudel can also stabilize active Cdc42 by sequestering a negative regulator, Cdc42GAP, at the leading edge during migration of NIH3T3 cells [28]. Nudel confers homodimerization and Lis1 binding through its N-terminal coiled-coil region, whereas its C-terminus is able to interact with dynein heavy chain, Cdc42GAP, and other proteins [23],[26],[28]–[30].

In this report, we describe a novel mechanism we identified that regulates adhesivity of integrin-mediated adhesions. Our results indicate that Nudel selectively strengthens FC sites in nascent adhesions through a direct interaction with paxillin to facilitate stabilization of membrane protrusions at the leading edge, whereas structurally activated FAK can displace Nudel from paxillin in a kinase-independent manner, thus reducing the strength of FC sites in FXs and FAs to promote retraction of the trailing side.

## Results

### Nudel Knockdown Impairs Nascent Cell Adhesion Independently of Rac1, Cdc42, and Dynein

**Figure 1:**
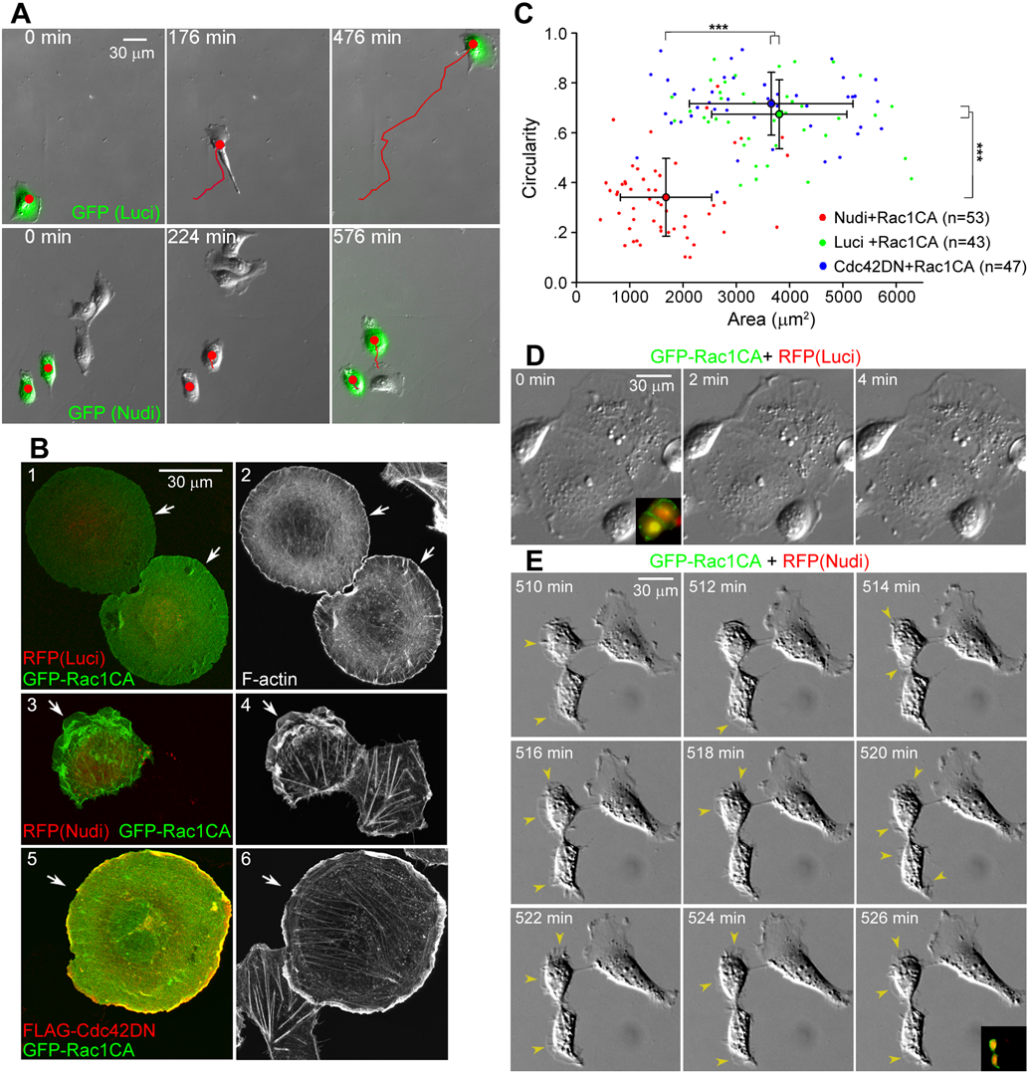
Nudel knockdown impairs cell spreading. (A) Nudel RNAi inhibits lamellipodial formation and cell migration. Representative image sequences are shown for ECV304 cells transfected with pTER-Nudi-GFP or pTER-Luci-GFP (green) for approximately 60 h. Red lines indicate cell tracks. See also Videos S1 and S2. (B and C) Nudel RNAi represses Rac1CA-stimulated cell spreading independently of Cdc42 activity. Panels 1–4: ECV304 cells were transfected for approximately 60 h with an indicated RNAi construct and then transfected again to express GFP-Rac1CA for approximately 12 h. Panels 5 and 6: cells were cotransfected for approximately 12 h to coexpress FLAG-Cdc42DN and GFP-Rac1CA. Arrows indicate transfectants. The area and circularity (4π×area/perimeter^2^) are used to reflect the extent of cell spreading. Error bars show SD. Asterisks indicate *p*<0.005. (D and E) Time-lapse images of typical control or Nudel-depleted cells overexpressing GFP-Rac1CA. Membrane protrusions (arrowheads) fail to be stabilized upon Nudel RNAi. Transfectants were identified through their coexpression of both RFP and GFP (insets). See also Videos S3 and S4.

We have previously shown that Nudel knockdown markedly inhibited pseudopodial formation in mouse fibroblast NIH3T3 cells [28]. To clarify whether this is solely related to defects in membrane protrusion, human epithelial ECV304 cells were chosen for analysis because they migrated with typical fan-shaped lamellipodia (Figure 1A; Videos S1 and S2). For convenient identification of live transfectants, the interference RNA (RNAi) constructs, pTER-Nudi for Nudel and pTER-Luci as a control [31], were modified to coexpress green fluorescent protein (GFP) or red fluorescent protein (RFP). As in NIH3T3 cells [28], Nudel RNAi in sparse ECV304 cells significantly repressed membrane protrusions and thus migration (Figures 1A, S1A, and S1B; Videos S1 and S2). Overexpression of Nudel with an RNAi-resistant construct (Nudel-R) rescued both lamellipodial formation and cell motilities (Figure S1C–S1E), thereby excluding a possible off-target effect of the RNAi construct.

Nudel RNAi has been shown to cause inactivation of Cdc42 [28], which could in turn repress Rac1 activity [32],[33]. If the lack of lamellipodia in Nudel-depleted cells (Figure 1A) was simply due to inhibition of Rac1, introduction of a constitutive active form of Rac1 (Rac1CA) should be able to fully restore lamellipodium formation [3],[34]. Consistent with a previous report [34], 76% of GFP-Rac1CA–positive cells cotransfected with pTER-Luci-RFP (*n* = 233) became flat and circular in shape, due to extensive formation and spreading of lamellipodia (Figure 1B, panels 1 and 2). In contrast, although 67% of pTER-Nudi-RFP transfectants overexpressing GFP-Rac1CA (*n* = 316) formed lamellipodia, as judged by the existence of F-actin–rich membrane ruffles, they failed to spread extensively (Figure 1B, panels 3 and 4, arrows). Quantitation also indicated that they generally exhibited obviously reduced circularity and area as compared to control cells (Luci+Rac1CA) (Figure 1C). To corroborate these results, we applied a dominant-negative Cdc42 (Cdc42DN) to repress Cdc42 activity (unpublished data) [35] and found that as expected, its overexpression failed to repress cell spreading stimulated by Rac1CA (Figure 1B and 1C). Therefore, the spreading defect associated with Nudel depletion is not solely due to inhibition of Cdc42 and Rac1.

We then performed time-lapse microscopy to examine why Nudel-depleted cells failed to fully spread even in the presence of Rac1CA. The control transfectants, which were much larger in size than surrounding untransfected cells, showed vigorous membrane ruffling at cell edges (Figure 1D; Video S3) [34]. In contrast, although GFP-Rac1CA induced active membrane protrusions in Nudel-depleted cells (Figure 1E vs. 1A), the protrusions were not persistent and usually retracted back within a few minutes (Figure 1E; Video S4), indicating lack of stable attachment to the substratum. As a result, the cells failed to spread even when monitored for more than 500 min (Figure 1E; Video S4).

We further excluded the possibility that Nudel RNAi repressed lamellipodial formation through inhibition of dynein because Nudel^C36^, a deletion mutant whose overexpression inhibits dynein [22],[23], failed to affect ECV304 cell migration (Figure S2A and S2B). Normal lamellipodial formation was seen as well in cells overexpressing either GFP-tagged Nudel^C36^ or another dynein inhibitor, p50^dynamitin^ (Figure S2C) [36],[37].

Taken together, these results strongly suggest a critical role of Nudel in stable attachment of nascent membrane protrusions to the substratum. Importantly, such a role is distinct from the previous ones in regulation of Cdc42 and dynein [28], therefore defining a novel function of Nudel in cell migration.

### Nudel RNAi Reduces the Efficiency of Nascent Adhesion Stabilization

**Figure 2 pbio-1000116-g002:**
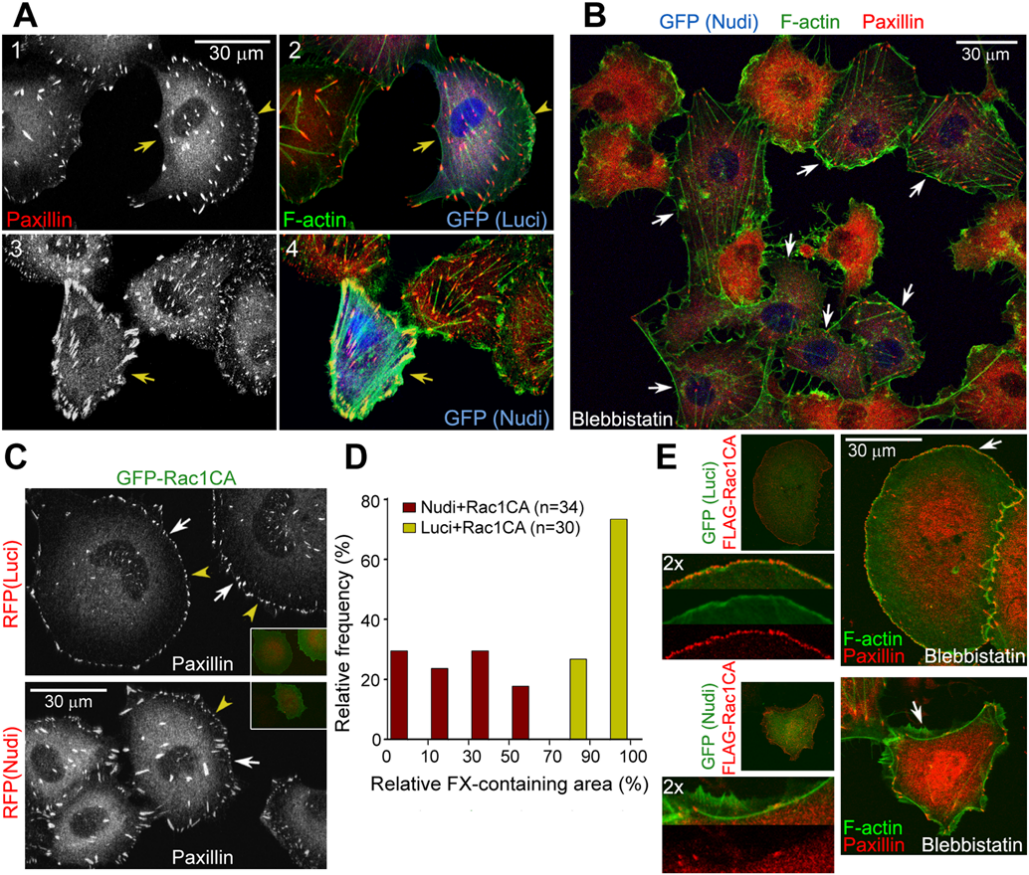
Effects of Nudel knockdown on FCs and stress fibers. (A and B) Nudel RNAi results in large peripheral FAs associated with thick stress fibers due to the collapse of cell edges (A). ECV304 cells were transfected with an indicated RNAi plasmid for approximately 72 h (arrows). Arrowheads indicate lamellipodia. In (B), cells were treated with 20 µM blebbistatin for 45 min to abolish myosin II-mediated tensions on stress fibers. More stress fibers and FAs were preserved in Nudel-depleted cells (arrows) after the drug treatment. As a result, untransfected cells generally contained higher levels of free paxillin and thus exhibited stronger cytosolic paxillin staining. (C and D) Nudel depletion attenuates FX formation even in the presence of GFP-Rac1CA. ECV304 cells were transfected as described in Figure 1B. Arrowheads indicate typical FXs. Relative FX-containing area was calculated as the percentage of radians of the FX-containing sector in a circle, centered at the centroid of a cell using ImageJ. (E) Nudel is important for nascent adhesion formation. ECV304 cells were transfected as described in Figure 1B and treated with 20 µM blebbistatin for 25 min prior to fixation to block the transition of nascent adhesions to FXs [5]. For four-color staining, F-actin was decorated with phalloidin-TRITC, whereas secondary antibodies conjugated with Alexa-405 and 647 were used with rabbit anti-FLAG antibody and anti-paxillin mAb to label FLAG-Rac1CA and paxillin. GFP was visualized through its autofluorescence. A typical region (arrow) was magnified to show details.

We then examined detailed distributions of FCs and F-actin in ECV304 cells with Nudel knockdown. Indeed, compared to the typical arc-like lamellipodial formation in most sparse transfectants of pTER-Luci-GFP (71.0%; *n* = 356) (Figure 2A, panels 1 and 2), transfection with pTER-Nudi-GFP resulted in severe cell edge shrinkage in both subconfluent cells (63.1%; *n* = 388) and confluent cells scratched to induce migration [38],[39] (Figures 2A, panels 3 and 4, and S3A). Moreover, robust FAs and stress fibers at the cell periphery were seen (Figures 2A, panels 3 and 4, and S3A). Similar phenotypes were also observed in HeLa cells, independent of cell densities (Figure S3B).

The FAs/stress fibers can develop in response to forces provided either intrinsically through contraction of myosin on stress fibers or externally by mechanical strains [12],[17],[18],[40]. To better understand the phenotypes of Nudel RNAi, we disrupted the intrinsic contractile forces using blebbistatin, a small-molecule inhibitor of myosin II ATPase activity [41]. After blebbistatin treatment for 45 min, FAs and stress fibers were mostly disassembled in control cells, as expected (Figure 2B) [5],[41]. Nevertheless, they were still largely preserved in Nudel RNAi cells (Figure 2B), suggesting that the robust FAs/stress fibers in Nudel-depleted cells (Figures 2A and S3) were formed in response to tensions from the collapsing cell edges in order to resist further shrinkage, instead of from the contractile forces of myosin II.

To understand why cell edges tended to shrink upon Nudel RNAi, we examined FCs in Nudel-depleted cells overexpressing Rac1CA. In control cells, Rac1CA induced typical FX around the entire cell periphery (Figure 2C and 2D) [34]. In contrast, although FXs were readily observed in pTER-Nudi-RFP transfectants overexpressing Rac1CA, they only appeared in less than half of the cell periphery in approximately 82% of cells (Figure 2C and 2D), indicating a markedly reduced efficiency of FX formation. We then treated such cells with blebbistatin for 25 min to block maturation of their nascent adhesions into FXs [5]. In contrast to the appearance of a rim of tiny, dense nascent adhesions within the lamellipodium in control cells (Figure 2E) [5], Nudel RNAi cells overexpressing Rac1CA showed little accumulation of nascent adhesions around the cell periphery (Figure 2E), though vigorous membrane protrusions still occurred (Figure 2E) as in untreated cells (Figure 1B, panels 3 and 4, and 1E). Therefore, the negative effect of Nudel RNAi on stabilization of membrane protrusions (Figures 1 and 2A) is attributed to poor formation of nascent adhesions.

### Nudel Directly Interacts with Paxillin

**Figure 3:**
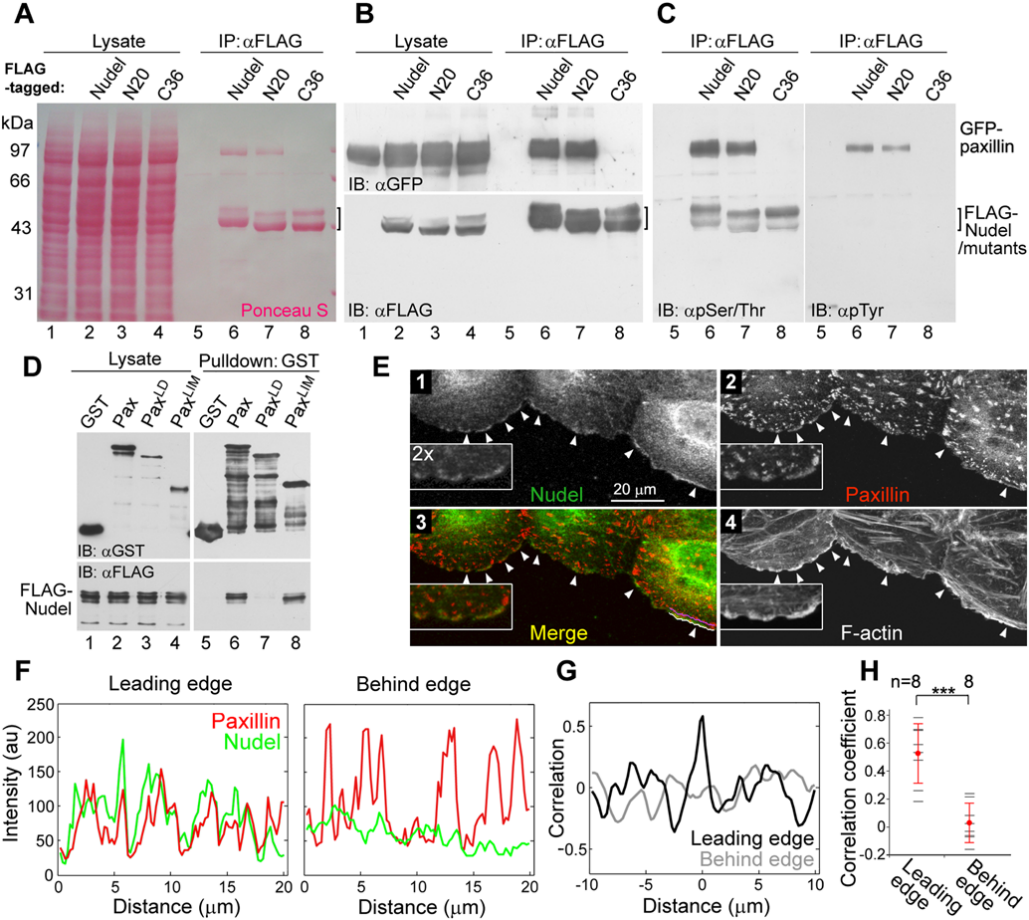
Interaction of Nudel with Paxillin. (A–C) Co-IP was performed with HEK293T cell lysates containing GFP-paxillin and an indicated FLAG fusion protein. After SDS-PAGE and transfer, the membrane was stained with Ponceau S prior to immunoblotting (IB) with the indicated antibodies. Nudel or mutants usually display two bands due to phosphorylation in mitotic cells [61]. Hyperphosphorylated Nudel (the upper band) was preferentially recognized by anti-phospho-Ser/Thr antibody (C). (D) Direct interaction of Nudel with paxillin in vitro. FLAG-Nudel, GST, and GST-tagged paxillin or mutants were expressed in *E. coli*. Bacterial lysates containing the indicated proteins were mixed and precipitated with glutathione beads. A diagram for paxillin mutants is presented in Figure S4A. (E) Nudel colocalizes with paxillin at regions of membrane protrusion. ECV304 monolayers cultured overnight in serum-free medium were scratched and then incubated for 3 h in the presence of serum. Enlargements are included to show details. Arrowheads indicate representative positions of membrane protrusion. (F) Fluorescent intensity of paxillin (red) and Nudel (green) along the white line (Leading edge) and the purple line (Behind edge) in (E), panel 3. (G) Cross-correlation analyses on data in (F) indicate a strong correlation between the intensity trace of Paxillin and Nudel at the leading edge and no correlation approximately 2 µm behind the edge. (H) Significant correlation of Nudel with paxillin at the leading edge. Statistic analyses were done using correlation coefficients obtained from eight different regions (arrowheads) in (E) as demonstrated in (F and G). Error bars show SD. Asterisks indicate *p*<0.005.

To understand how Nudel could affect nascent adhesions, we performed a screen for its partner(s) in FCs. FLAG-Nudel coexpressed with a GFP-tagged FC protein such as vinculin, paxillin, or FAK was subjected to coimmunoprecipitation (co-IP). Ponceau S staining revealed GFP-paxillin as the major protein associated with FLAG-Nudel (Figure 3A and 3B, lane 6), strongly suggesting a direct interaction. GFP-paxillin was also associated with FLAG-Nudel^N20^, a mutant lacking Lis1-binding activity [22], but not with FLAG-Nudel^C36^ (Figure 3A and 3B, lanes 7 and 8). The failure of Nudel^C36^ to interact with paxillin was also consistent with the results that, unlike the wild-type Nudel (Figure S1D and S1E), Nudel^C36^ overexpressed from an RNAi-resistant construct failed to restore the motility of Nudel-depleted cells (Figure S2A and S2B). FLAG-Nudel was able to associate with Tyr/Ser/Thr-phosphorylated isoforms important for physiological functions of paxillin (Figure 3C) [42]–[44], further suggesting a functional interplay between the two proteins.

To confirm their direct interaction, GST-paxillin and FLAG-Nudel were expressed in *Escherichia coli*. Glutathione S-transferase (GST)-pulldown assays indeed indicated their interaction (Figure 3D, lane 6). Moreover, when paxillin mutants containing either the LD domains or the LIM domains (Figure S4A) [15] were assayed, only Pax^LIM^ interacted with Nudel (Figure 3D, lanes 8). Reciprocal experiments also support a direct Nudel-paxillin interaction (Figure S4B). In contrast, vinculin, a paxillin-associated FC protein [15], failed to bind directly to Nudel (Figure S4B). As paxillin exists in all types of FCs and is a scaffold/adaptor protein critical for cell migration [6],[15], it may serve as the target of Nudel in cell adhesion. Consistently, FLAG-Nudel formed a complex with endogenous paxillin and vinculin (see below).

We then examined localization of Nudel and paxillin in ECV304 cells migrating into an artificial “wound” [38],[39]. As in NIH3T3 cells [28], Nudel was enriched at the leading edge, and colocalized with paxillin there (Figure 3E, arrowheads). Moreover, both proteins were enriched in areas of cell protrusions, indicated by the presence of active actin polymerization (Figure 3E) [4],[45]. In contrast, Nudel did not show colocalization with the paxillin puncta, which typically represent FXs and FAs (Figure 3E) [46]. Quantitation analyses also indicated a significant correlation between Nudel and paxillin at the leading edge (Figure 3F–3H). These results imply interaction of both proteins in early stages of FC formation and are consistent with the role of Nudel in nascent membrane adhesion (Figures 1 and 2).

### Forced Localization of Nudel with Paxillin in FCs Enhances Integrin-Mediated Adhesions

**Figure 4:**
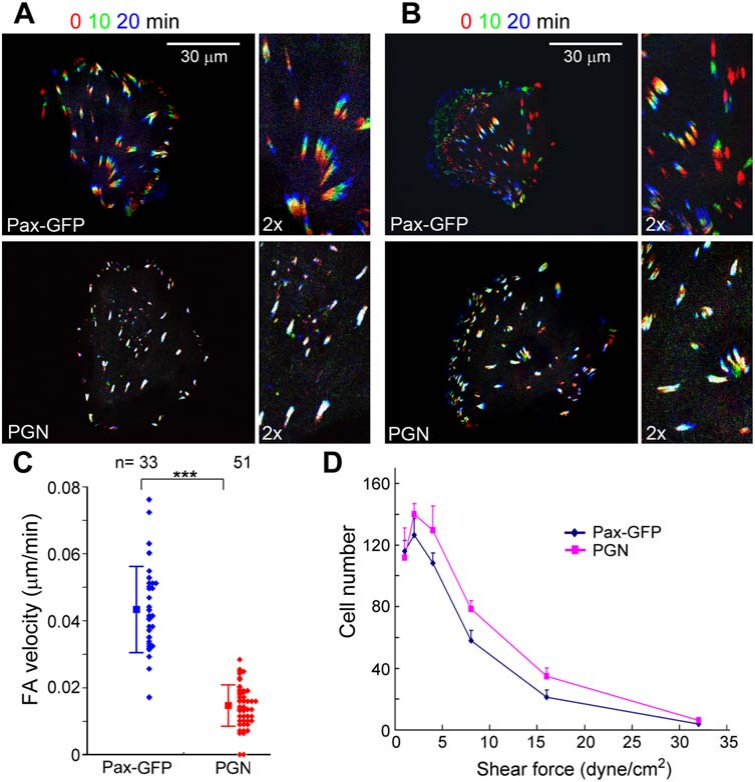
Overexpression of PGN strengthens integrin-mediated adhesion. (A–C) PGN reduces FA motilities. ECV304 cells were transfected to express paxillin (Pax)-GFP or PGN (the paxillin-GFP-Nudel fusion protein). GFP fluorescence of live cells was recorded at 5-min intervals. Triple color overlay of image sequences are used to show FA motilities within 20 min. ECV304 cells in (A) were immotile during imaging, whereas those in (B) were migrating. Individual FA velocity was calculated from the track length in 50 min and is presented as a solid diamond in (C). Error bars show SD. Asterisks indicate *p*<0.005. (D) Cell adhesivities on laminin and fibronectin substrates are increased. HEK293T cells overexpressing Pax-GFP or PGN were infused into flow chamber. The total number of cells remaining adherent at each indicated wall shear stress was determined. See Materials and Methods for details. Error bars show SD (three experiments).

We then tried to assess whether the Nudel-paxillin interaction indeed contributed positively to nascent cell adhesion. As integrin-mediated nascent adhesion sites are submicroscopic structures and only represented a portion of total adhesion sites (Figures 2 and 3) [7], direct assays on them would not be feasible. We thus reasoned that a fusion protein, paxillin-GFP-Nudel (PGN), would make all adhesion sites Nudel-containing, thus allowing convenient examination of Nudel's effect on adhesion. Although such a construct is somewhat artificial, a similar strategy has been successfully used in other studies [47]. Similar to Pax-GFP (Figure 4A and 4B) [48], PGN was also located in FCs (Figures 4A, 4B, S5A, and S5B). Moreover, PGN still bound to Lis1 (Figure S5C), a protein associated with the N-terminal portion of Nudel [30]. Therefore, both paxillin and Nudel in the fusion protein are still functional.

To verify whether PGN stabilized the cell–substratum adhesion, we first examined FA motilities [19], which may reflect the stability of individual adhesion sites of FAs against tension. For easy comparison, image sequences at 0, 10, and 20 min were pseudocolored red, green, and blue, respectively, and merged. Motile FAs would thus display rainbow colors, whereas nonmotile ones would be white [19]. Upon overexpression of Pax-GFP, FAs in both nonmotile (Figure 4A) and motile cells (Figure 4B) exhibited similar active centripetal movement, as judged by the appearance and orientation of rainbow colors. The average velocity was 0.0434 µm/min (Figure 4C), about 3-fold lower than that of 3T3 fibroblasts [19]. It should be noted that Smilenov and colleagues [19] considered cells just after division as “migrating” cells and defined the remaining population as “stationary” cells. Therefore, the population analyzed herein is equivalent to the “stationary” population in the previous study [19]. Just as in ECV304 cells (Figure 1), this population of fibroblasts is in fact not truly stationary [28].

In cells overexpressing PGN, FA motilities were largely reduced, as judged by the obvious appearance of white color (Figure 4A and 4B). The average velocity of FAs was reduced by approximately 3-fold (0.0146 µm/min) as compared to that in cells overexpressing Pax-GFP (Figure 4C). Moreover, as FCs close to the cell edges where membranes are dynamic showed obvious turnover in PGN-positive cells as well (Figure 4B), the reduced FA motility is unlikely due to defects in FA disassembly. Rather, it suggests an increased strength of the FC sites.

To further corroborate the above results, we investigated whether PGN-positive cells exhibited enhanced adhesion on a laminin- and fibronectin-coated surface against different shear forces [49]. HEK 293T cells were used instead of ECV304 because the latter cells tended to aggregate, thus precluding sorting with fluorescence-activated cell sorter (FACS) to eliminate untransfected cells. At the wall shear stress of 1 and 2 dyne/cm^2^, PGN-expressing cells accumulated more rapidly than Pax-GFP–positive cells (Figure 4D). They maintained a higher resistance to the increasing shear stress from 2 to 16 dyne/cm^2^ (Figure 4D).

Taken together, these results strongly suggest that the Nudel-paxillin association can enhance adhesion strength of FC sites. This further explains why Nudel is critical for stabilization of nascent membrane protrusions (Figures 1 and 2).

### FAK Sequesters Paxillin from Nudel through Physical Interaction

**Figure 5:**
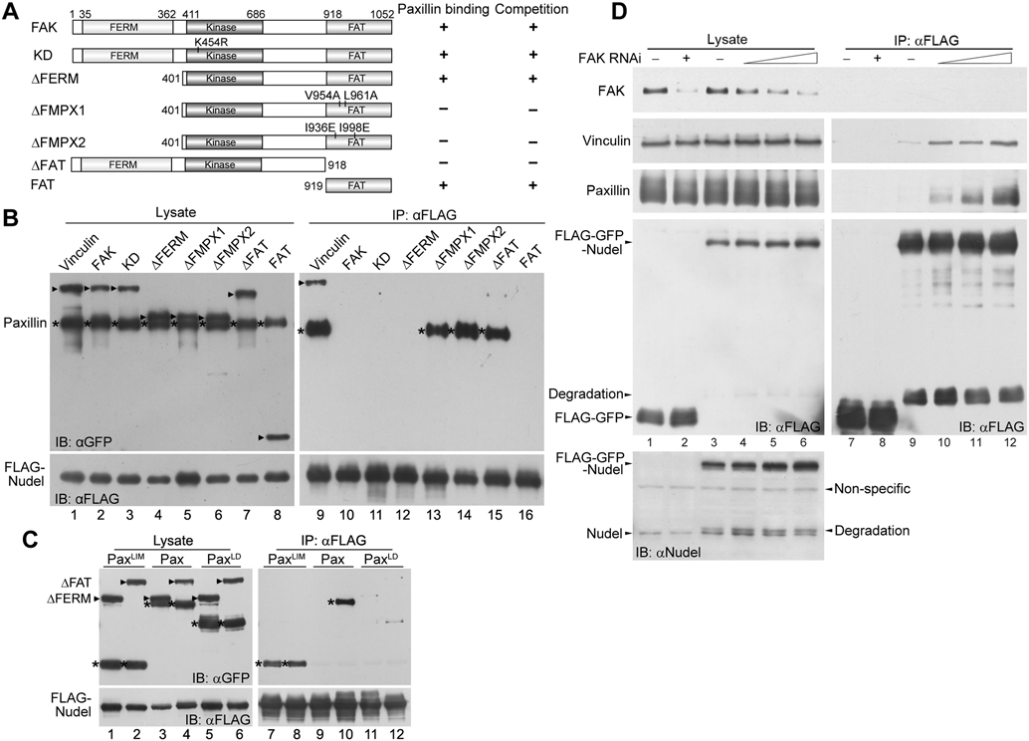
FAK competes against Nudel for paxillin. (A) Schematic diagrams of FAK and mutants. Their paxillin-binding abilities are summarized from literature [51],[52]. Their abilities to compete with Nudel for paxillin are based on results in (B). (B) Overexpression of FAK or certain mutants disrupts the Nudel-paxillin interaction. Co-IP was performed with lysates of HEK293T cells overexpressing FLAG-Nudel, GFP-Paxillin (asterisks), and a GFP fusion protein as indicated above (arrowheads). (C) Nudel associates with the LIM domain of paxillin, and the interaction is not affected by FAK. Co-IP was performed with lysates of HEK293T cells overexpressing FLAG-Nudel, GFP-tagged FAK^ΔFERM^ or FAK^ΔFAT^ (arrowheads), and GFP-tagged paxillin or a mutant indicated above (asterisks). (D) Regulation of the Nudel-paxillin interaction by endogenous FAK. Low levels of FLAG-GFP-Nudel (at 3–6-fold of the endogenous Nudel level) or FLAG-GFP were expressed in HEK293T cells transfected with either control or FAK RNAi plasmids (lanes 1–6). After co-IP, proteins associated with anti-FLAG resin were visualized by immunoblotting.

As Nudel was not seen in either FXs or FAs (Figure 3E), we speculated that it might be displaced by certain paxillin-binding protein(s) that are recruited during the maturation of nascent adhesion sites [6]. Indeed, co-IP results indicated that overexpression of GFP-tagged FAK, but not vinculin, both of which are paxillin-binding FC proteins [15], abolished the interaction between GFP-paxillin and FLAG-Nudel (Figure 5A and 5B, lanes 1 and 2 vs. 9 and 10). FAK mutants lacking the paxillin-binding FAT domain [2], e.g., FAK^ΔFAT^ and FAK^Kin^, were completely ineffective (Figure 5A and 5B, lane 7 vs. 15; Figure S6A and S6B, lane 1 vs. 4). In contrast, mutants containing FAT or even FAT alone, e.g., FAK^ΔFERM^, FAK^FAT^, and FRNK, a naturally occurring FAK isoform [50], were all potent in disrupting the Nudel-paxillin interaction (Figure 5A and 5B, lanes 4 and 8 vs. 12 and 16; Figure S6A and S6B, lanes 2 vs. 5). When the paxillin-interacting ability of FAK^ΔFERM^ was abolished by point mutations V954A/L961A [51] or I936E/I998E [52], the resultant mutants, FAK^ΔFMPX1^ and FAK^ΔFMPX2^, failed to disrupt the Nudel-paxillin interaction (Figure 5A and 5B, lanes 5 and 6 vs. 13 and 14). In contrast, the kinase-dead mutant FAK^KD^ carrying a K454R mutation [53] still showed strong activity in competing for paxillin binding (Figure 5A and 5B, lane 3 vs. 11). Therefore, FAK can, in a kinase-independent manner, disrupt the Nudel-paxillin interaction through its physical association with paxillin.

To understand how FAK abolishes the Nudel-paxillin interaction, GFP-tagged Pax^LD^ and Pax^LIM^ (Figure S4A) [15] were tested for their ability to bind Nudel in the presence of GFP-FAK^ΔFAT^ or FAK^ΔFERM^ (Figure 5C). As expected (Figure 5A and 5B), the Nudel-paxillin interaction was not affected by FAK^ΔFAT^ but was disrupted by FAK^ΔFERM^ (Figure 5C, lanes 3 and 4 vs. 9 and 10). Pax^LIM^ associated with Nudel in both cases (Figure 5C, lanes 1 and 2 vs. 7 and 8), whereas Pax^LD^ failed to do so in either case (Figure 5C, lanes 5 and 6 vs. 11 and 12). These data further confirm that Nudel interacts with paxillin via the LIM domains (Figure 3D). Moreover, competition by FAK is mediated through its direct interaction with the LD domains of paxillin [2],[15].

To further investigate whether FAK indeed regulates the Nudel-paxillin interaction at physiological conditions, we performed co-IP experiments to check whether a decrease in endogenous FAK levels could affect the Nudel-paxillin interaction. As we were not able to detect endogenous paxillin in co-IP experiments using anti-Nudel IgY, possibly due to a steric effect of the antibody, we overexpressed in HEK293T cells low levels of FLAG-GFP-Nudel (at 3–6-fold of endogenous Nudel level) through the internal ribosome entry site (IRES) and performed co-IP assays with anti-FLAG resin (Figure 5D). The levels of endogenous FAK were reduced sequentially through transfection of increasing amounts of the RNAi plasmids (Figure 5D, lanes 1–6). Indeed, association of endogenous paxillin with FLAG-GFP-Nudel was markedly enhanced following the reduction of endogenous FAK levels (Figure 5D, lanes 7–12). Association of vinculin was also detected, with its levels paralleling those of paxillin (Figure 5D, lanes 7–12). Therefore, endogenous FAK can regulate the Nudel-paxillin interaction as well.

### FAK Overexpression Induces Cell Edge Collapse in a Paxillin-Binding– and Open Conformation–Dependent Fashion

**Figure 6:**
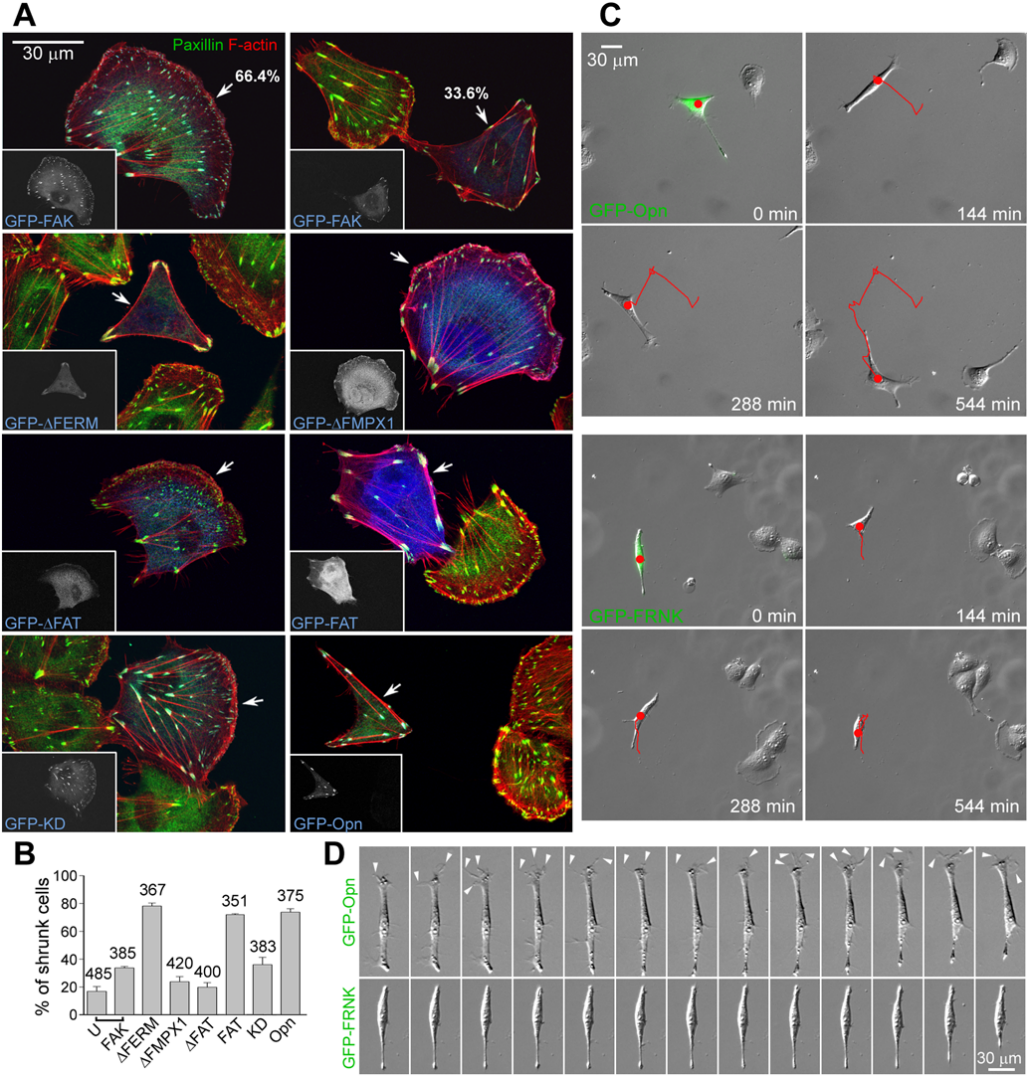
Effects of FAK and mutants on ECV304 cell adhesion. (A and B) Typical morphologies of ECV304 cells overexpressing GFP-tagged FAK or its mutants (arrows). Only GFP-FAK^FAT^ lacked FA localization. Incidences of polygonal cells are shown in the histogram (B). U, cells negative for GFP-FAK in the same population. Error bars show SD. (C and D) FAK^Opn^ and FRNK have different effects on cell migration and membrane protrusion. Images of live ECV304 cells overexpressing GFP-FAK^Opn^ or GFP-FRNK (green) are shown in (C) together with cell tracks (red). Representative frames of 2-min intervals are presented in (D) for comparison. Dynamic membrane protrusions at the front are indicated by arrowheads. See also Videos S5 and S6. Statistical data for cell motility are shown in Figure S6D.

If the Nudel-paxillin interaction was indeed critical for nascent membrane adhesion, according to the above results (Figure 5A and 5B), overexpressing FAK or any FAK mutant that binds paxillin should displace Nudel prematurely from nascent adhesion sites and consequently impair cell spreading. Indeed, overexpression of any FAT-containing deletion mutant, i.e., FAK^ΔFERM^, FRNK, or even FAK^FAT^, resulted in high incidences (≥72%) of cell shrinkage: affected cells usually lacked FXs and were typically polygonal in shape, with cell edges supported by F-actin bundles and FAs (Figures 6A, 6B, and S6C). In contrast, mutants lacking the FAT domain, e.g., FAK^ΔFAT^ and FAK^Kin^, or containing FAT but lacking paxillin-binding activity, e.g., FAK^ΔFMPX1^ and FAK^ΔFMPX2^, only generated background levels of shrunken cells (Figures 6A, 6B, and S6C). Moreover, although FAK^ΔFAT^ and FAK^Kin^ failed to show FA localization, FAK^ΔFMPX1^ was still efficiently targeted to FAs (Figures 6A and S6C) [51]. FAK^ΔFMPX2^ exhibited weak, but clear, FA localization as well (Figure S6C). Because FAK^ΔFMPX1^ and FAK^ΔFMPX2^ do not bind to paxillin, their localization to FA is probably mediated by talin [2],[51],[52],[54]. Thus, the paxillin-binding activity of FAK is essential for the induction of cell edge shrinkage, whereas its kinase domain is dispensable.

In contrast to the intact FAT-containing mutants, full-length FAK only showed a mild effect. Although GFP-FAK–positive cells with the shrinkage phenotypes were approximately 2-fold higher in percentages than surrounding untransfected cells, the majority of cells overexpressing GFP-FAK (66.4% in average) showed normal lamellipodia (Figure 6A and 6B). The kinase-dead mutant GFP-FAK^KD^ had a similar effect (Figure 6A and 6B), whereas FAK^Opn^, a mutant containing two point mutations (Y180A/M183A) that abrogate the autoinhibitory effect of the FERM domain [55] potently induced cell edge shrinkage upon its overexpression (Figure 6A and 6B). Similar phenotypes were seen in ECV304 cells grown on fibronectin- and/or laminin-coated substratum as well as in CV1 and NIH3T3 cells (Figure S7; unpublished data). Therefore, the “open” structure of full-length FAK is important for both full activation of FAK [55] and induction of cell edge shrinkage.

FAK is believed to promote cell migration through its kinase activity [2]. To understand why both FAK^Opn^, which exhibits robust kinase activity in cells (unpublished data) [55], and FRNK, which is a dominant-negative mutant on kinase activity of endogenous FAK [50], caused similar cell shrinkage phenotypes (Figure 6A and 6B), we monitored behaviors of live cells. Consistent with results in fixed cells (Figure 6A and 6B), ECV304 cells overexpressing GFP-FAK^Opn^ were narrow or polygonal in shape (Figure 6C; Video S5). Whereas surrounding untransfected cells migrated through typical arc-like lamellipodia, these transfectants extended long processes rich in transient filopodium-like projections (*n* = 19/20) and migrated like fibroblasts (Figure 6C and 6D; Video S5) [28]. In contrast, cells overexpressing GFP-FRNK showed markedly reduced motilities (Figures 6C and S6D; Video S6). Such cells also failed to show active membrane protrusions (*n* = 21/21) (Figure 6D), consistent with the lack of FAK kinase activity. Therefore, although cells overexpressing FAK^Opn^ or FRNK showed different motilities, they share similar shrinkage phenotypes.

These results identify a novel kinase-independent role of FAK in cell spreading. As this role of FAK depends on its interaction with paxillin, the “shrunken” phenotype caused by FAK overexpression is attributed to poor adhesions of nascent membrane protrusions due to premature disruption of the Nudel-paxillin interaction at the leading edge.

## Discussion

### Nudel Is Critical for Both Membrane Protrusion and Its Subsequent Stabilization

We have previously shown that Nudel is required for membrane protrusions in NIH3T3 cells [28]. Here, we further showed that Nudel depletion markedly repressed lamellipodial formation in ECV304 cells (Figure 1A; Video S2), indicating a general requirement of Nudel in membrane protrusion. Lamellipodial formation requires Rac activity, whereas Cdc42 can activate Rac [3],[32],[34]. Therefore, the protrusion defect upon Nudel depletion is consistent with inhibition of Cdc42 activity [28]. By contrast, although Nudel is also essential for dynein functions [22],[23], dynein activity is not important for lamellipodial formation and free migration of ECV304 cells (Figure S2).

Nudel is also critical for stabilization of membrane protrusions by facilitating nascent adhesion formation. First, Nudel depletion by RNAi primarily resulted in cell edge collapse (Figures 2A and S3). As the robust stress fibers in Nudel RNAi cells were not sensitive to blebbistatin treatment (Figure 2B), their formation is unlikely due to increased contractile forces on stress fibers, e.g., through activation of Rho GTPase [34],[40]. Rather, it is attributed to mechanical strains caused by cell edge shrinkage because stress fibers induced by mechanical forces do not depend on myosin II activity [18],[40],

Second, although overexpression of Rac1CA rescued the membrane protrusion defect of Nudel depletion, cells still failed to fully spread due to poor adhesions of their protruded membranes (Figure 1B and 1E; Video S4). Importantly, this phenotype is not caused by the inactivation of Cdc42 per se, as coexpression of a dominant-negative form of Cdc42 with Rac1CA failed to repress cell spreading (Figure 1B and 1C).

Third, the markedly reduced formation of FXs as well as nascent adhesions upon Nudel depletion even in the presence of Rac1CA (Figure 2C and 2E) further indicates a positive role of Nudel in nascent adhesions.

### Nudel Stabilizes Nascent Adhesions through Interaction with Paxillin at the Leading Edge

The Nudel-paxillin interaction further substantiated the role of Nudel in nascent adhesion because paxillin can bind directly to integrin and is thus one of the earliest intracellular proteins at nascent adhesions [2],[5],[6]. As the interaction between exogenous Nudel and paxillin was readily detected even by Ponceau S staining after co-IP (Figure 3A), these two proteins appear to interact with high affinity. Moreover, they interacted directly through their C-terminal domains (Figures 3 and S4). The interaction between endogenous paxillin and FLAG-Nudel expressed at a relative low level can also be detected in vivo, especially upon knockdown of FAK expression (Figure 5D).

Our results suggest that Nudel interacts with paxillin in nascent adhesions. Complex formation of Nudel with both paxillin and vinculin (Figures 5B, lane 9, and 5D) suggests its localization in certain FCs. Nevertheless, it was not detected in FXs or FAs, but enriched and colocalized with paxillin at the leading edge in areas of active membrane protrusions (Figure 3E–3H), where nascent adhesions occur [4],[5]. Moreover, a localization of Nudel in nascent adhesions is also consistent with its functions there (Figures 1 and 2).

We provided evidence showing that the presence of Nudel in FCs can indeed stabilize integrin-ECM interactions using PGN (Pax-GFP-Nudel) (Figures 4 and S5). PGN was specifically located in FCs (Figure S5B) and reduced FA motilities by approximately 3-fold, compared to Pax-GFP (Figure 4A–4C), suggesting elevated stability, or strength, of individual adhesion site. Furthermore, the elevated adhesiveness of PGN-positive cells over Pax-GFP-positive ones, measured through their resistance to shear forces (Figure 4D) [49], further supports the increase in adhesion strengths. Although PGN is an artificial protein and may not precisely reflect situations in vivo, its effects on FA motility and cell adhesion are well in agreement with other results that suggest a role of Nudel in stabilization of nascent adhesions (Figures 1–3). Consistently, paxillin-deficient cells exhibit a delayed rate of spreading [56]. Nevertheless, it is currently not known whether the Nudel-paxillin interaction stabilizes integrin-ECM ligations by modulating the integrin conformation or by regulating other intracellular adhesion molecules.

### Structurally Activated FAK Represses Nascent Adhesions by Disrupting the Nudel-Paxillin Interaction

We demonstrated that FAK is a key regulator of the Nudel-paxillin interaction. FAK was able to disrupt the interaction via direct binding to paxillin (Figure 5). Such a competition effect may be mediated through steric hindrance. Alternatively, given that the FAT domain alone, which covers only one-eighth of FAK, was already sufficient to disrupt the Nudel-paxillin interaction (Figure 5A and 5B), FAK binding may induce in paxillin a conformational change that abrogates Nudel binding. That FAK and Nudel bound to distinct regions of paxillin (Figures 3D and 5C) [15] also supports the latter speculation.

In addition to its known kinase-dependent functions in cell migration [2], we found that FAK can negatively regulate nascent adhesions. Overexpression of FAK resulted in an approximately 2-fold increase in incidence of cells with shrunken edges comparing to surrounding untransfected populations (Figure 6A and 6B). Deleting the FERM domain (e.g., FAK^ΔFERM^) or abolishing its autoinhibitory role through point mutations (e.g., FAK^Opn^) [55] considerably augmented incidences of the shrunken phenotype (Figure 6A and 6B). Such a phenotype, however, is not correlated with the kinase activity of FAK because it is similar in cells overexpressing either the hyperactive (e.g., FAK^Opn^ and FAK^ΔFERM^) [55] or the dominant-negative (e.g., FRNK and possibly FAK^FAT^) [50] mutants (Figures 6A, 6B, and S6C). In addition, in the absence of the FERM domain, the potency of FAK to induce cell edge collapse is only correlated with its interaction with paxillin (Figures 6A, 6B, and S6C). Localization of FAK in FCs, however, is not sufficient: the point mutants FAK^ΔFMPX1^ and FAK^ΔFMPX2^ localized to FAs but failed to cause the shrunken phenotype (Figures 6A and S6C).

**Figure 7:**
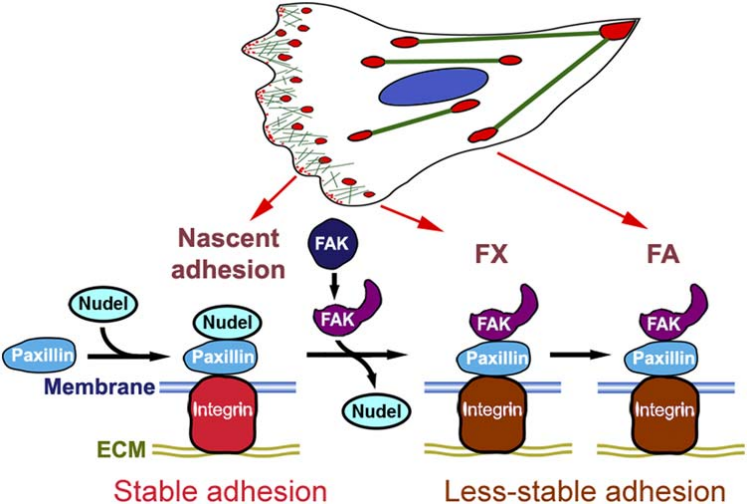
A summarizing model. In migrating cells, interaction of Nudel with paxillin at nascent adhesion sites stabilizes the integrin-ECM ligation to promote adhesion of membrane protrusions. When the autoinhibitory effect of the FERM domain of FAK is abrogated by signaling events [55], the FAT domain is exposed and able to displace Nudel from paxillin. Adhesion sites in both FXs and FAs are Nudel-free and thus exhibit reduced adhesion strength. Such a change in FAs may facilitate FA motilities and retraction of the trailing side.

We therefore propose a model to explain how paxillin, Nudel, and FAK cooperate to modulate integrin-mediated adhesivity in cell migration (Figure 7): during membrane protrusion, activated integrin molecules located on polymerizing F-actin [4] bind to ECM to form nascent adhesion sites containing paxillin [6]; association of Nudel with paxillin strengthens such sites; upon formation of the open conformation in response to external signals, possibly through interaction of integrin and/or growth factor receptors [55], FAK displaces Nudel from paxillin; adhesion sites now exhibit a lower strength than those containing Nudel.

The antagonizing roles of Nudel and FAK in adhesivity provide a mechanism for cells to properly coordinate adhesion and migration. The positive effect of Nudel on adhesion strength can stabilize nascent adhesion sites and thus facilitate stabilization of membrane protrusions at the leading edge. Stronger adhesiveness would also allow nascent sites to transmit stronger traction forces [13] and to resist retraction. On the other hand, because FAs are large in size, a decreased strength of their individual FC sites would facilitate FA movement (Figure 4) [19] and retraction of the trailing side.

Our findings also help to understand how cells orchestrate different events in migration. As formation of the open structure of FAK depends on upstream signals and serves as a prerequisite for activation of the kinase [55], disruption of the Nudel-paxillin interaction, thus down-regulation of adhesivity at nascent adhesions, is likely to precede other events associated with the kinase activity of FAK [2]. Such an ordered sequence of action appears important for cell migration because premature disruption of the Nudel-paxillin interaction and/or interference with the kinase activity of FAK affects cell motility. For instances, although excess wild-type FAK failed to interfere with lamellipodial formation in the majority of cells (Figure 6A and 6B), overexpression of FAK^Opn^ to prematurely disrupt the Nudel-paxillin interaction (Figures 5, 6, and S6) while provoking a hyperactive kinase activity [55], impaired the arc-like lamellipodium formation in ECV304 cells and resulted in cell migration through transient filopodium-like membrane projections (Figure 6; Video S5). In contrast, overexpression of FRNK to similarly abrogate the Nudel-paxillin interaction (Figure S6) while also inhibiting endogenous FAK activity [50],[57] caused cell shrinkage but poor migration (Figures 6 and S6; Video S6) [57]. Furthermore, FAK-null cells have been shown to exhibit robust FC formation at the cell periphery [58],[59], reminiscent of enhanced cell edge adhesions to the substratum. These cells also show poor migration [58],[59].

We have previously shown that Nudel can stabilize active Cdc42 at the leading edge by sequestering Cdc42GAP in NIH3T3 cells [28]. Nudel also contributes to dynein functions at the leading edge [28],[60]. Moreover, similar to paxillin (Figure 3), both Cdc42GAP and dynein heavy chain bind to the C-terminus of Nudel [22],[28]. How these functions of Nudel are coordinated is not yet clear. One possibility is that Nudel interacts with different partners for different functions or in different cell types. Another possibility is that these partners use Nudel as a common platform to achieve orchestration of different functions. Interestingly, in co-IP experiments, we found that associations of Cdc42GAP, paxillin, and dynein with Nudel were significantly enhanced upon overexpression of both paxillin and Cdc42GAP (Figure S8). If such a synergetic effect on Nudel binding occurred at the leading edge due to enrichment of these proteins there (Figure 3) [28],[60], the Nudel-paxillin interaction and the regional activation of Cdc42 and dynein would become spatiotemporally coupled events to eventually facilitate establishment of a polarized lamellipodium. These issues will be worthy of future investigations.

## Materials and Methods

### Plasmid Constructs

Expression plasmids for human Nudel, its mutants, and p50^dynamitin^ were described previously [22],[28],[61]. pLV-IRES-FLAG-GFP and pLV-IRES-FLAG-GFP-Nudel were constructed from a lentiviral vector (a gift from Qiwei Zhai, Institute of Nutritional Science, Shanghai Institutes for Biological Sciences [SIBS]) for low-level expression of FLAG fusion proteins via the internal ribosome entry site (IRES). pTER-Nudi, a Nudel RNAi construct, and a control construct pTER-Luci [31] were further modified to coexpress GFP or RFP. The RNAi-resistant Nudel constructs contained three silent mutations in the short hairpin RNA (shRNA)-target region. The expressed proteins, despite unchanged amino acid sequences, were named Nudel-R and Nudel^C36^-R sheer for presentation purposes. To silence FAK expression, pTER-FAKi1 and pTER-FAKi2 were constructed and cotransfected at a 1∶1 ratio. Their targeting sequences are 5′GGTACTGGTATGGAACGTTCT3′ and 5′GCCTTAACAATGCGTCAGT3′, respectively. Expression plasmid for GFP-vinculin was kindly provided by Benjamin Geiger (Weizmann Institute, Israel). pGFP-hPaxillin and pVSV-mFAK/FRNK were gifts from Kenneth M.Yamada (National Institute of Dental and Craniofacial Research [NIDCR], National Institutes of Health [NIH]). To express fusion proteins paxillin-GFP or Paxillin-GFP-Nudel, the coding sequence of paxillin was amplified by PCR and inserted in-frame between the NheI and AgeI sites of pEGFP-C1 or pEGFP-C1-Nudel. FAK and paxillin mutants were created by PCR as well. Plasmids for expression of GFP-tagged Rac1, FLAG-tagged Cdc42, and mutants were from Xiaobing Yuan (Institute of Neuroscience, SIBS) and Michiyuki Matsuda (Osaka University, Japan). Plasmids containing PCR fragments were subjected to sequencing confirmation.

### Antibodies and Staining Reagents

Mouse monoclonal antibodies (mAbs) to α-tubulin, vinculin, FLAG, and phospho-Tyr, and rabbit antibodies to FAK and FLAG were purchased from Sigma-Aldrich. mAbs to paxillin and phospho-Ser/Thr were from BD Biosciences Transduction Laboratories. Rabbit antibody to GFP was from Santa Cruz Biotechnology. Anti-GST mAb was from Wolwo Biotech. Anti-Nudel IgY was generated from chicken and affinity-purified [23]. Secondary antibodies conjugated with peroxidase or Alexa Fluor-405, -488, -546, or -647 were purchased from Invitrogen. Phalloidin-Alexa-647 was from Invitrogen. Phalloidin-TRITC and blebbistatin were from Sigma-Aldrich.

### Cell Culture, Transfection, and Sorting

All cells were cultured at 37°C and 5% CO_2_ in Dulbecco's modified Eagle's medium (Invitrogen) supplemented with 10% (v/v) bovine serum (Sijiqing). Human embryonic kidney (HEK) 293T cells were transfected by using the conventional calcium phosphate method. This cell line was used for assays involving immunoprecipitation due to its high transfection efficiency. Human bladder epithelial ECV304 [62] and cervical carcinoma HeLa cells were transfected with Lipofectamine2000 (Invitrogen). In overexpression experiments, cells were harvested approximately 48 h posttransfection for biochemical assays or fixed approximately 20 h posttransfection for microscopy. In RNAi experiments, they were used 48–72 h posttransfection. To determine RNAi efficiency in ECV304, GFP-positive transfectants were enriched to approximately 90% by using a BD FACSAria cell sorter 48 h after transfection. To prevent cell aggregation, Latrunculin A (0.1 µg/ml; Invitrogen) was added prior to sorting. Transfectants were cultured for an additional 24 h and then collected for immunoblotting (IB).

### Immunoprecipitation and Immunoblotting

Approximately 1×10^7^ HEK293T cells were lysed in co-IP buffer (20 mM Tris-HCl [pH 7.5], 100 mM KCl, 0.1% NP-40, 1 mM EDTA, 10% glycerol, 1 mM DTT, 50 mM NaF, 10 mM Na-pyrophosphate, 1 mM Na-Vanadate, and protease inhibitors cocktail [Calbiochem]) by repetitive pipetting through a 1-ml tip. After centrifugation at 10,000 *g* for 10 min to remove debris, lysates were incubated with anti-FLAG M2 agarose beads (Sigma) for 2 h on a rotator at 4 °C. The beads were then washed with the buffer for three times, followed by elution with synthetic FLAG peptide [63]. For pull-down assays, bacterial lysates containing GST fusion proteins or FLAG-Nudel were premixed for 1 h and then incubated with glutathione or anti-FLAG agarose beads (Sigma-Aldrich) for another 1 h at 4°C with agitation. Proteins binding to the beads were then boiled in SDS-sample buffer and subjected to IB. When necessary, membranes were stripped and blotted with different antibodies. Experiments were repeated at least three times.

### Fluorescence Staining and Confocal Microscopy

Unless indicated, cells were grown sparsely on sterile glass coverslips without pre-coating of ECM. They were fixed with 4% paraformaldehyde (Sigma-Aldrich) for 15 min, followed by permeabilization with 0.5% Triton X-100 (v/v) for 10 min. For scratch wound assays, confluent cell monolayers cultured in serum-free medium for 12 h were scratched with yellow tips [28] and then cultured in serum-containing medium for an additional 3 h prior to fixation.

Immunofluorescence staining was performed with appropriate combinations of antibodies. F-actin was decorated with fluorochrome-labeled phalloidin. Images were captured with a Leica TCS SP2 laser-scanning confocal microscope. Grayscale images were converted to pseudocolor using Adobe Photoshop. Statistical data were presented as mean±standard deviation (SD) from at least three experiments. Cell area and circularity (4π×area/perimeter^2^) were measured using ImageJ (NIH). To quantify fluorescent colocalizations along the leading edge, intensity profiles were obtained using ImageJ. Cross-correlations and Pearson correlation coefficients of the intensity profiles were calculated with Matlab (MathWorks) [4].

### Time-Lapse Microscopy

ECV304 cells were cultured in L-15 medium (Invitrogen) supplemented with 10% (v/v) bovine serum. Image sequences for cell migration were collected by using an Olympus IX81 microscope with 37 °C-incubation chamber, motorized stage, and Evolution QEi CCD camera (Media Cybernetics), or a Leica AS MDW workstation with a heating hood and a CoolSNAP HQ CCD camera (Roper Scientific) [28],[64]. For FA motility assays, cells were imaged by using an Olympus FluoView 1000 inverted confocal microscope with a heating stage at 5-min intervals. ImageJ (NIH) was used for measurement. Migration tracks were determined as tracks of nuclei [28]. Average velocity of a sparse cell was calculated using its track length of free migration.

### Flow Chamber Assays

Flow chamber assays were performed basically as described [49]. A polystyrene Petri dish coated with purified human laminin and fibronectin (12.5 µg/ml each; Sigma-Aldrich) was used as the lower wall of the chamber. HEK293T transfectants were trypsinized and sorted. GFP-positive cells were diluted to 1×10^6^/ml in complete culture medium and infused into the flow chamber immediately. Cells were allowed to accumulate for 30 s at 0.3 dyne/cm^2^ and for 10 s at 0.4 dyne/cm^2^. Shear stress was then increased every 10 s from 1 dyne/cm^2^ up to 32 dyne/cm^2^ in 2-fold increments. The number of cells remaining bound at the end of each 10-s interval was counted.

## Supporting Information

Figure S1 **Efficiency and specificity of Nudel RNAi.** (A) Characterizations of RNAi constructs. ECV304 cells transfected with pTER-Luci-GFP (lane 1) or pTER-Nudi-GFP (lane 2) for 3 d were sorted out by FACS based on GFP fluorescence, whereas HeLa cells were assayed directly after transfection with pTER (lane 3) or pTER-Nudi (lane 4). Immunoblotting was then performed to detect the indicated proteins. (B) Statistics of ECV304 cell motilities. T, transfectants; U, untransfected cells. Asterisks indicate *p*<0.01. Error bars show SD. Representative videos and images are in Figure 1A and Videos S1 and S2. (C) Validation of the RNAi-resistant Nudel construct (pEGFP-Nudel-R). HEK293T cells were cotransfected with the pTER-Nudi-RFP and a plasmid for expression of GFP-tagged Nudel or Nudel-R for 3 d. Lysates were then subjected to immunoblotting. (D and E) Overexpressing GFP-Nudel-R in pTER-Nudi-RFP transfectants rescues cell migration. Image sequences of live pTER-Nudi-RFP transfectants overexpressing GFP or GFP-Nudel-R (yellow) are presented with cell tracks (red lines). In the statistics, error bars show SD. Asterisks indicate *p*<0.005.
(0.87 MB TIF)

Figure S2 **Autonomous migration of ECV304 cells is independent of dynein activity.** (A and B) Image sequences of typical ECV304 cells overexpressing the indicated proteins. Overexpression of GFP-Nudel^C36^ inactivates cytoplasmic dynein [22],[23] but had little effect on random migration of ECV304 cells. In Nudel RNAi cells, however, GFP-Nudel^C36^ expressed from an RNAi-resistant construct (pEGFP-Nudel^C36^-R) was unable to restore cell migration. In the statistics (B), error bars are SD. Asterisks indicate *p*<0.005. (C) ECV304 cells overexpressing GFP-Nudel^C36^ or GFP-p50 (arrows) still form normal lamellipodia (arrowheads). p50 is a dynactin subunit whose overexpression inactivates dynein as well [36],[37].
(1.75 MB TIF)

Figure S3 **Phenotypes of Nudel RNAi in HeLa and scratched ECV304 cells.** (A) ECV304 cells transfected with pTER-Luci-GFP or pTER-Nudi-GFP for three days were scratched as described [28] and fixed after 3 h. Arrows indicate transfectants located at wound edges. (B) HeLa cells were transfected with pTER-Luci-GFP or pTER-Nudi-GFP for 3 d. Arrows point to representative transfectants. Merged images were enlarged to show details. Panels 5–12 show morphologies of cells growing in different densities.
(3.06 MB TIF)

Figure S4 **Interaction of Nudel with paxillin.** (A) Schematic diagrams of paxillin (Pax) and mutants. (B) Interaction of Nudel with paxillin, but not vinculin, in vitro. Bacterial lysates containing GST-tagged vinculin or paxillin were mixed with lysates containing FLAG-Nudel and then subjected to co-IP with anti-FLAG resin. This experiment is reciprocal to that in Figure 3D.
(0.14 MB TIF)

Figure S5 **Characterization of the paxillin-GFP-Nudel fusion protein (PGN).** (A) Validation of PGN. HEK293T cells were transfected to overexpress the indicated proteins. Immunoblotting (IB) was then performed with the indicated antibodies. PGN was recognized by antibodies against GFP, Nudel, and paxillin, respectively. (B) Localization of PGN in FCs in ECV304 cells. Vinculin is used as a marker for FCs. Transfectants are indicated by arrows. (C) Nudel in PGN still interacts with Lis1. FLAG-Lis1 was overexpressed with the indicated GFP-fusion proteins in HEK293T cells. Co-IP and immunoblotting were then performed.
(0.51 MB TIF)

Figure S6 **Effects of some FAK mutants on Nudel-paxillin interaction and cell adhesion.** (A) Schematic diagrams of FAK mutants. Their abilities to bind paxillin or to compete with Nudel for paxillin are summarized on the right. (B) Co-IP was performed with lysates of HEK293T cells coexpressing FLAG-Nudel, GFP-Paxillin, and an indicated GFP-FAK mutant (arrowheads). Similar results were obtained in NIH3T3 and ECV304 cells (unpublished data). (C) Typical morphologies of ECV304 cells overexpressing the indicated GFP-FAK mutant (arrows). Incidences of the shrinkage phenotypes are shown in the histogram. (D) Statistics for motilities of the indicated cell populations. Errors show SD. Asterisks indicate *p*<0.005. Representative cell images are shown in Figure 6C.
(1.28 MB TIF)

Figure S7 **FAK^Opn^ overexpression induces cell shrinkage in different cell lines.** CV1, NIH3T3, or ECV304 cells were transfected to express either GFP-tagged FAK^Opn^ or FAK^ΔFAT^. ECV304 cells were plated on glass coverslips coated with fibronectin (12.5 µg/ml) and laminin (12.5 µg/ml) to examine the influence of ECM on FAK^Opn^-induced cell shrinkage.
(3.14 MB TIF)

Figure S8 **Synergetic effect of paxillin, Cdc42GAP, and dynein on Nudel binding.** The indicated exogenous proteins were expressed separately in HEK293T cells. Their cell lysates were premixed as indicated in lanes 1–5 for 2 h and then subjected to co-IP with anti-FLAG resin (lanes 6–10). GFP-Cdc42GAP associated with FLAG-Nudel is indicated by arrowheads in the top panel. In the bottom panel, the uppermost band represents phosphorylated Nudel or Nudel^C36^ [61]. DIC, dynein intermediate chain.
(0.30 MB TIF)

Video S1 **Migration of a typical ECV304 cell transfected with pTER-Luci-GFP.** The cell was monitored at 2-min intervals for 476 min. The first and last frames are GFP fluorescence images. Representative frames are shown in Figure 1A. Scale bar indicates 30 µm.
(1.43 MB MOV)

Video S2 **Nudel RNAi by pTER-Nudi-GFP impairs cell migration.** Transfectants were monitored for 576 min at 2-min intervals. The first and last frames are GFP fluorescence images. Representative frames are shown in Figure 1A. Scale bar indicates 30 µm.
(2.69 MB MOV)

Video S3 **Extensive spreading of control ECV304 cells overexpressing GFP-Rac1CA.** pTER-Luci-RFP transfectants overexpressing GFP-Rac1CA were monitored for 398 min at 2-min intervals. The first and second frames are RFP and GFP images, respectively. Representative frames are shown in Figure 1D. Scale bar indicates 30 µm.
(2.46 MB MOV)

Video S4 **pTER-Nudi-RFP transfectants overexpressing GFP-Rac1CA failed to spread.** Cells were monitored for 528 min at 2-min intervals. The first and second frames are RFP and GFP images, respectively. Representative frames are shown in Figure 1E. Scale bar indicates 30 µm.
(2.02 MB MOV)

Video S5 **Migration of ECV304 cells overexpressing GFP-FAK^Opn^.** Cells were monitored for 544 min at 2-min intervals. The first frame shows GFP fluorescence. Representative frames are shown in Figure 6C and 6D. Scale bar indicates 30 µm.
(4.75 MB MOV)

Video S6 **Migration of ECV304 cells overexpressing GFP-FRNK.** Cells were monitored for 544 min at 2-min intervals. The first frame shows GFP fluorescence. Representative frames are shown in Figure 6C and 6D. Scale bar indicates 30 µm.
(2.90 MB MOV)

## Abbreviations

co-IP: coimmunoprecipitation
ECM: extracellular matrix
FA: focal adhesion
FAT: FA-targeting
FC: focal contact
FX: focal complex
RNAi: RNA interference
SD: standard deviation
