# Supplementary figures and images for: Nudel and FAK as Antagonizing Strength Modulators of Nascent Adhesions through Paxillin
Source: PLoS Biol. 2009 May 26;7(5):e1000116. doi: 10.1371/journal.pbio.1000116 (PMC2684528; doi:10.1371/journal.pbio.1000116)

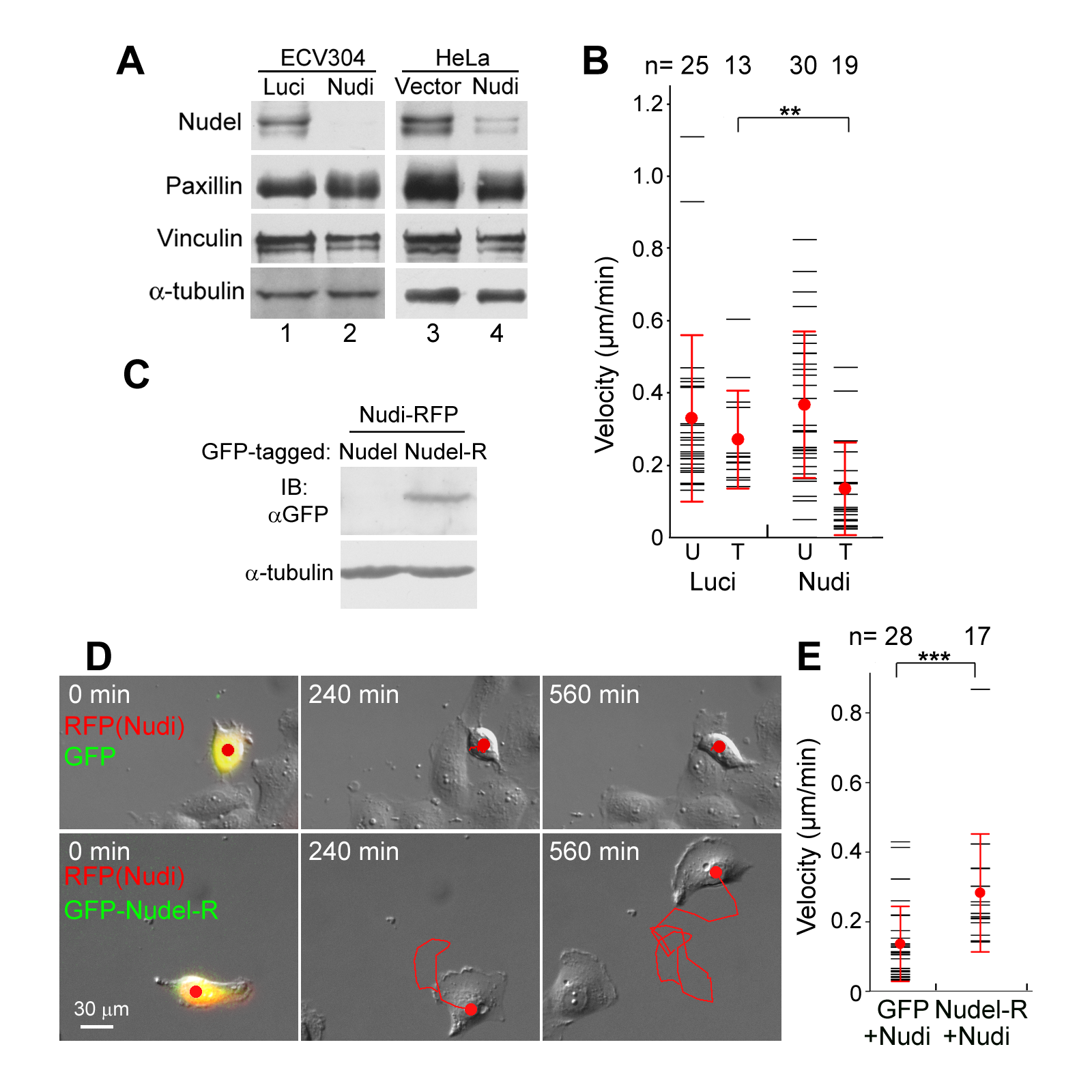

Supplement: Figure S1 — Efficiency and specificity of Nudel RNAi. (A) Characterizations of RNAi constructs. ECV304 cells transfected with pTER-Luci-GFP (lane 1) or pTER-Nudi-GFP (lane 2) for 3 d were sorted out by FACS based on GFP fluorescence, whereas HeLa cells were assayed directly after transfection with pTER (lane 3) or pTER-Nudi (lane 4). Immunoblotting was then performed to detect the indicated proteins. (B) Statistics of ECV304 cell motilities. T, transfectants; U, untransfected cells. Asterisks indicate p<0.01. Error bars show SD. Representative videos and images are in Figure 1A and Videos S1 and S2. (C) Validation of the RNAi-resistant Nudel construct (pEGFP-Nudel-R). HEK293T cells were cotransfected with the pTER-Nudi-RFP and a plasmid for expression of GFP-tagged Nudel or Nudel-R for 3 d. Lysates were then subjected to immunoblotting. (D and E) Overexpressing GFP-Nudel-R in pTER-Nudi-RFP transfectants rescues cell migration. Image sequences of live pTER-Nudi-RFP transfectants overexpressing GFP or GFP-Nudel-R (yellow) are presented with cell tracks (red lines). In the statistics, error bars show SD. Asterisks indicate p<0.005. (0.87 MB TIF) [file pbio.1000116.s001.tif]

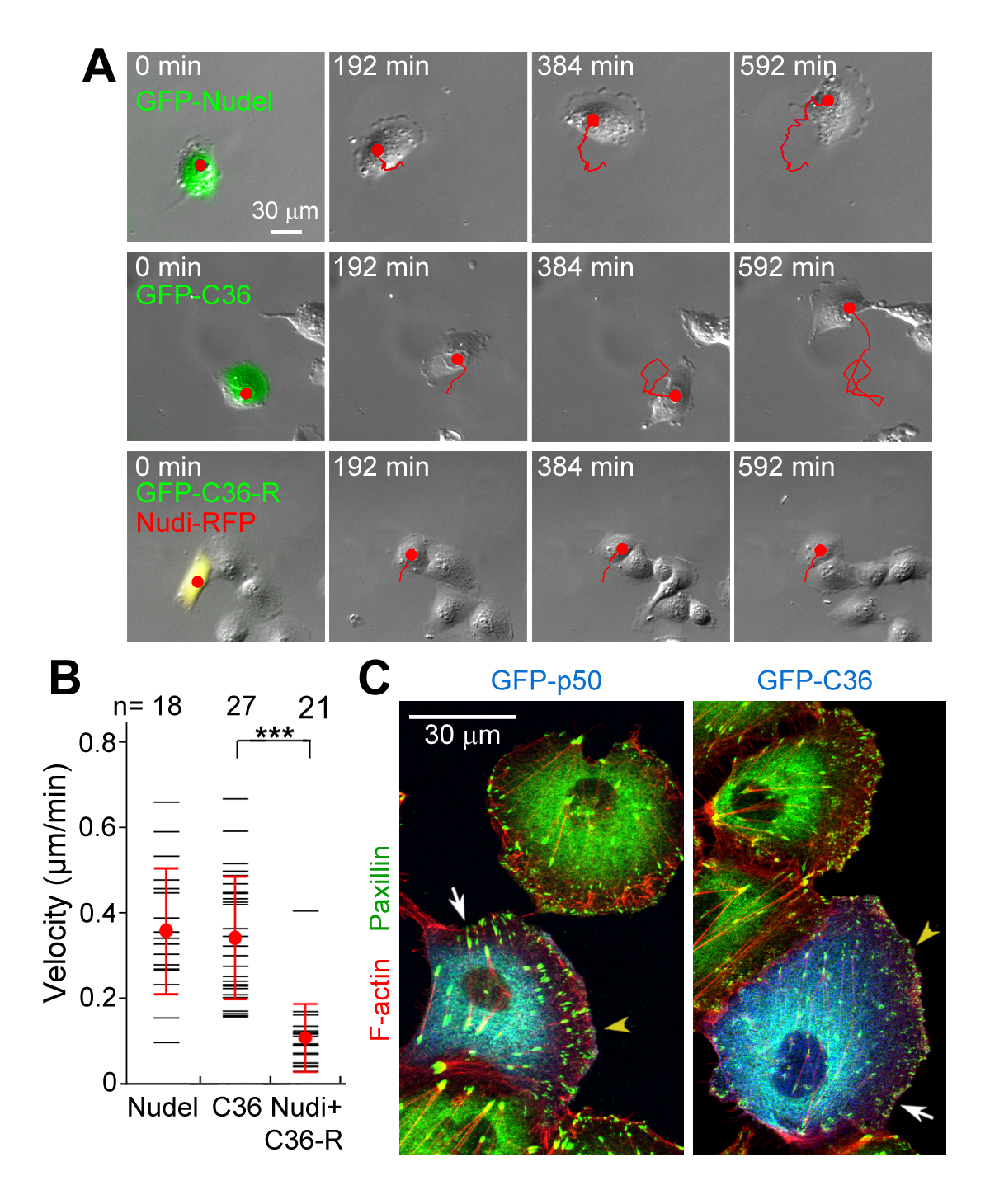

Supplement: Figure S2 — Autonomous migration of ECV304 cells is independent of dynein activity. (A and B) Image sequences of typical ECV304 cells overexpressing the indicated proteins. Overexpression of GFP-NudelC36 inactivates cytoplasmic dynein [22],[23] but had little effect on random migration of ECV304 cells. In Nudel RNAi cells, however, GFP-NudelC36 expressed from an RNAi-resistant construct (pEGFP-NudelC36-R) was unable to restore cell migration. In the statistics (B), error bars are SD. Asterisks indicate p<0.005. (C) ECV304 cells overexpressing GFP-NudelC36 or GFP-p50 (arrows) still form normal lamellipodia (arrowheads). p50 is a dynactin subunit whose overexpression inactivates dynein as well [36],[37]. (1.75 MB TIF) [file pbio.1000116.s002.tif]

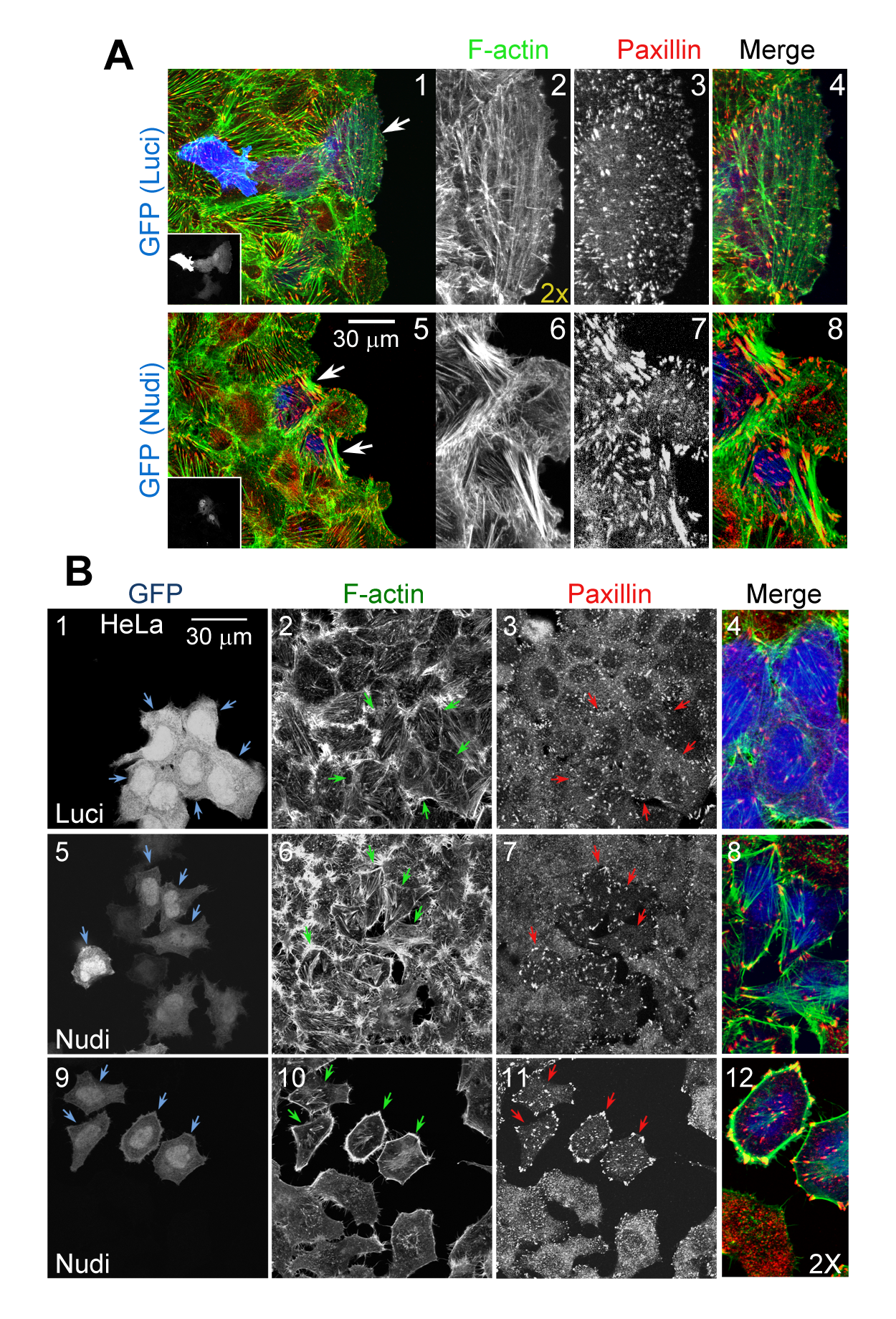

Supplement: Figure S3 — Phenotypes of Nudel RNAi in HeLa and scratched ECV304 cells. (A) ECV304 cells transfected with pTER-Luci-GFP or pTER-Nudi-GFP for three days were scratched as described [28] and fixed after 3 h. Arrows indicate transfectants located at wound edges. (B) HeLa cells were transfected with pTER-Luci-GFP or pTER-Nudi-GFP for 3 d. Arrows point to representative transfectants. Merged images were enlarged to show details. Panels 5–12 show morphologies of cells growing in different densities. (3.06 MB TIF) [file pbio.1000116.s003.tif]

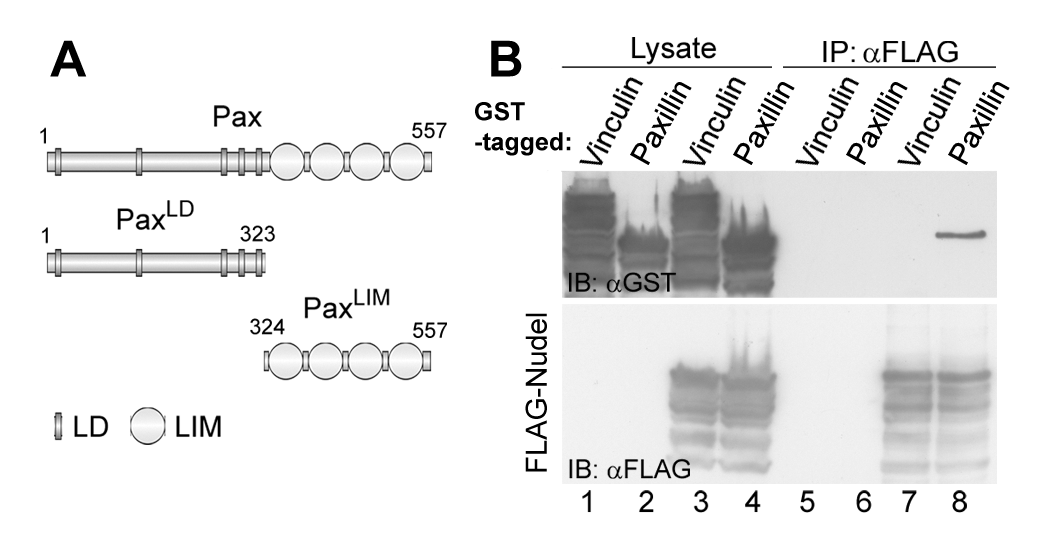

Supplement: Figure S4 — Interaction of Nudel with paxillin. (A) Schematic diagrams of paxillin (Pax) and mutants. (B) Interaction of Nudel with paxillin, but not vinculin, in vitro. Bacterial lysates containing GST-tagged vinculin or paxillin were mixed with lysates containing FLAG-Nudel and then subjected to co-IP with anti-FLAG resin. This experiment is reciprocal to that in Figure 3D. (0.14 MB TIF) [file pbio.1000116.s004.tif]

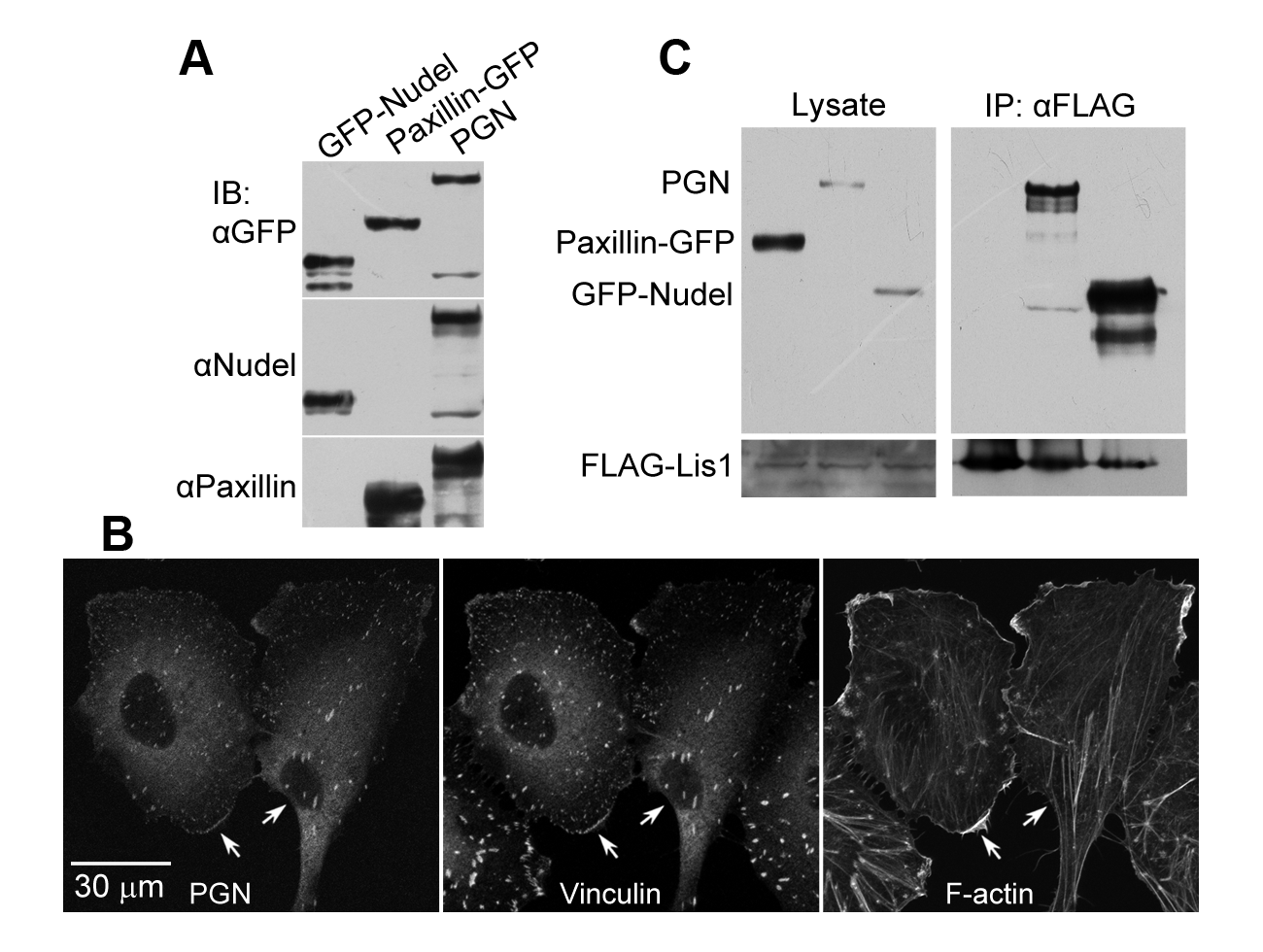

Supplement: Figure S5 — Characterization of the paxillin-GFP-Nudel fusion protein (PGN). (A) Validation of PGN. HEK293T cells were transfected to overexpress the indicated proteins. Immunoblotting (IB) was then performed with the indicated antibodies. PGN was recognized by antibodies against GFP, Nudel, and paxillin, respectively. (B) Localization of PGN in FCs in ECV304 cells. Vinculin is used as a marker for FCs. Transfectants are indicated by arrows. (C) Nudel in PGN still interacts with Lis1. FLAG-Lis1 was overexpressed with the indicated GFP-fusion proteins in HEK293T cells. Co-IP and immunoblotting were then performed. (0.51 MB TIF) [file pbio.1000116.s005.tif]

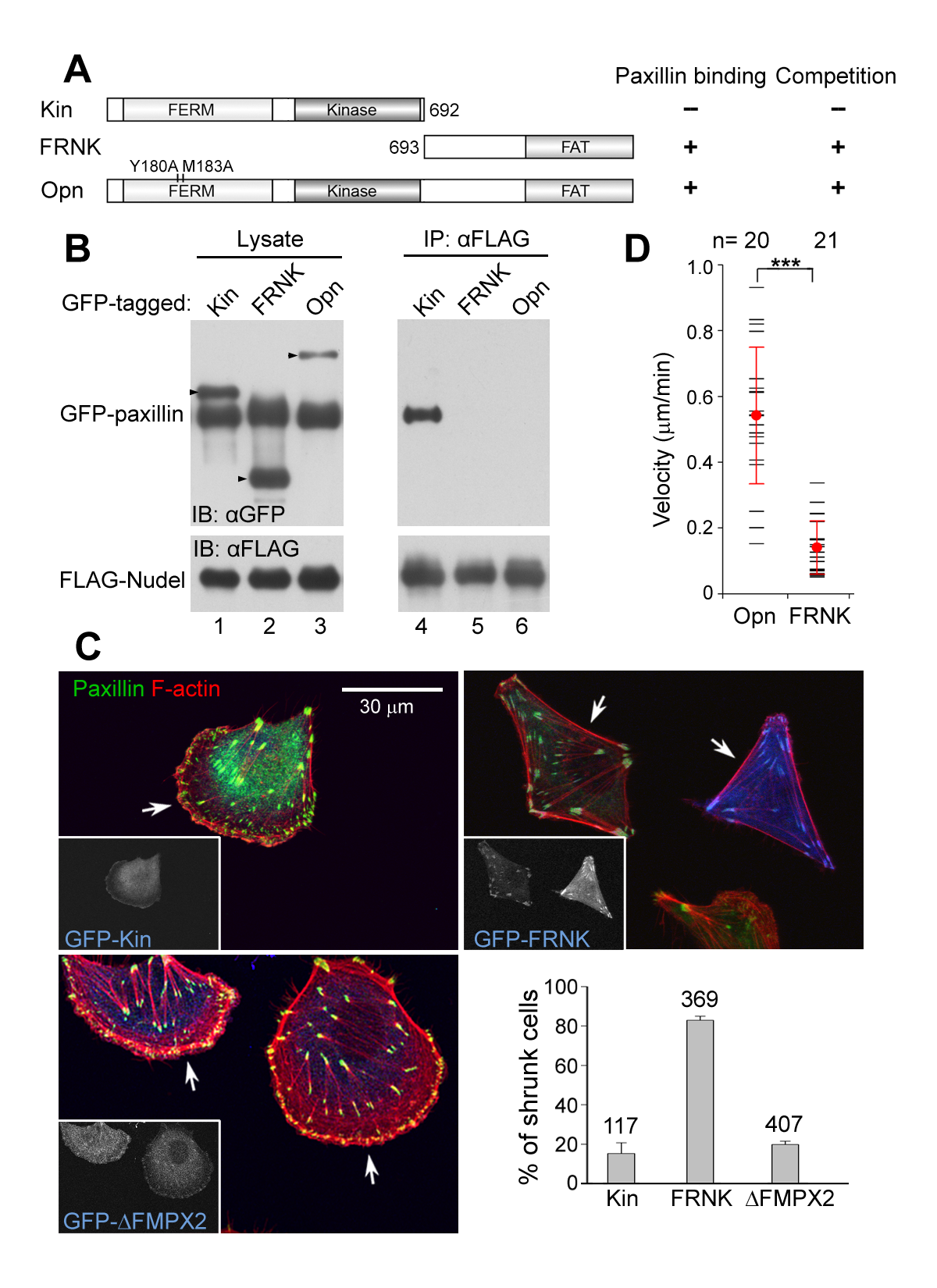

Supplement: Figure S6 — Effects of some FAK mutants on Nudel-paxillin interaction and cell adhesion. (A) Schematic diagrams of FAK mutants. Their abilities to bind paxillin or to compete with Nudel for paxillin are summarized on the right. (B) Co-IP was performed with lysates of HEK293T cells coexpressing FLAG-Nudel, GFP-Paxillin, and an indicated GFP-FAK mutant (arrowheads). Similar results were obtained in NIH3T3 and ECV304 cells (unpublished data). (C) Typical morphologies of ECV304 cells overexpressing the indicated GFP-FAK mutant (arrows). Incidences of the shrinkage phenotypes are shown in the histogram. (D) Statistics for motilities of the indicated cell populations. Errors show SD. Asterisks indicate p<0.005. Representative cell images are shown in Figure 6C. (1.28 MB TIF) [file pbio.1000116.s006.tif]

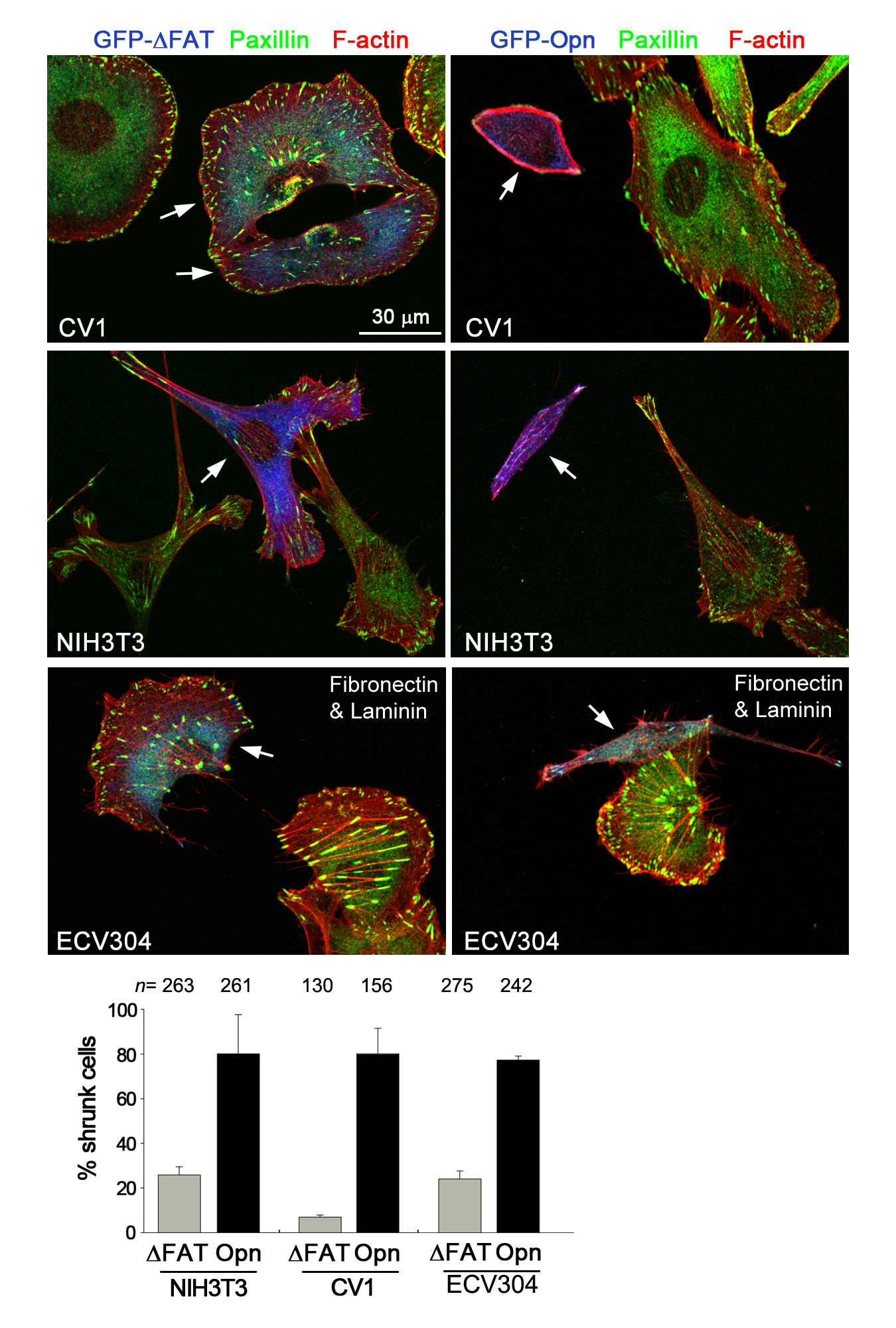

Supplement: Figure S7 — FAKOpn overexpression induces cell shrinkage in different cell lines. CV1, NIH3T3, or ECV304 cells were transfected to express either GFP-tagged FAKOpn or FAKΔFAT. ECV304 cells were plated on glass coverslips coated with fibronectin (12.5 µg/ml) and laminin (12.5 µg/ml) to examine the influence of ECM on FAKOpn-induced cell shrinkage. (3.14 MB TIF) [file pbio.1000116.s007.tif]

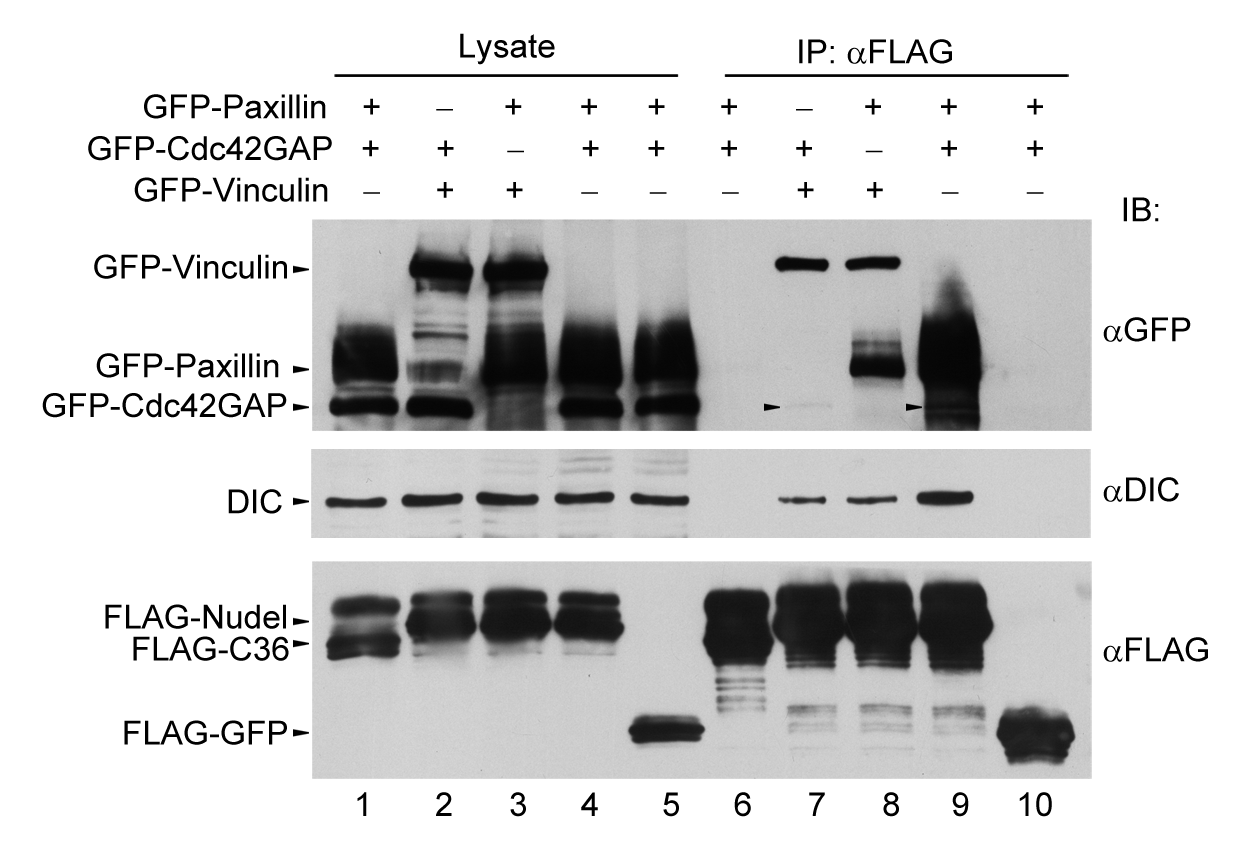

Supplement: Figure S8 — Synergetic effect of paxillin, Cdc42GAP, and dynein on Nudel binding. The indicated exogenous proteins were expressed separately in HEK293T cells. Their cell lysates were premixed as indicated in lanes 1–5 for 2 h and then subjected to co-IP with anti-FLAG resin (lanes 6–10). GFP-Cdc42GAP associated with FLAG-Nudel is indicated by arrowheads in the top panel. In the bottom panel, the uppermost band represents phosphorylated Nudel or NudelC36 [61]. DIC, dynein intermediate chain. (0.30 MB TIF) [file pbio.1000116.s008.tif]
